# Supplementary material for: Aptamer-guided delivery of miR-126 suppresses glioblastoma stem-like cell invasion and tumor progression
Source: Mol Ther Nucleic Acids. 2026 Sep 17;37(4):103103. doi: 10.1016/j.omtn.2026.103103 (PMC13639097; doi:10.1016/j.omtn.2026.103103)
Supplement: Supplementary file 1 [file mmc1.pdf]

**OMTN, Volume *37***

## Supplemental information

# Aptamer-guided delivery of miR-126 suppresses glioblastoma stem-like cell invasion and tumor progression

**Giorgia Castellani, Mariachiara Buccarelli, Alessandra Affinito, Gabriele De Luca, Ramona Ilari, Francesca Pedini, Alessandra Di Giamberardino, Mariagiovanna Ballato, Gabriele Ricciardi, Martina Offi, Cristiana Mollinari, Nadia Felli, Alessandra Boe, Giovanna Marziali, Mauro Biffoni, Maria Caffo, Gerolama Condorelli, Maurizio Martini, Quintino Giorgio D'Alessandris, and Lucia Ricci-Vitiani**

**Table S1.** Clinical features of GBM patients and tumors.

| Variable | n=58 |
|---|---|
| **Age (y)** | |
| - median | 63.0 |
| - mean ±sd | 61.8 ± 11.9 |
| **Sex n (%)** | |
| - male | 36 (62.1%) |
| - female | 22 (37.9%) |
| **Tumor location n (%)** | |
| - frontal | 23 (39.6%) |
| - temporal | 18 (31%) |
| - parietal | 11 (19%) |
| - occipital | 3 (5.2%) |
| - other | 3 (5.2%) |
| **Ki67 expression n (%)** | |
| - <20 | 19 (32.8%) |
| - ≥20 | 35 (60.3%) |
| - N/A | 4 (6.9%) |
| **MGMT status n (%)** | |
| - Methylated | 30 (51.7%) |
| - Unmethylated | 11 (19%) |
| - N/A | 17 (29.3%) |
| **KPS n (%)** | |
| - **≤70** | 18 (31%) |
| - **>70** | 40 (69%) |

Abbreviations: MGMT, O6-methylguanine-DNA-methyltransferase; KPS, Karnofsky performance status.

**Table S2.** Scheme of the intracranial implantation of GSCs into immunocompromised mice and aptamer-based treatment protocol.

| Group | n | Treatment (Dose) | Schedule | Administration method |
|---|---|---|---|---|
| I | 10 | saline | Three times/week (Three Weeks) | i.v. (tail vein) |
| II | 10 | A40s (2500 pmol) | Three times/week (Three Weeks) | i.v. (tail vein) |
| III | 10 | A40s-miR-126-3p (2500 pmol) | Three times/week (Three Weeks) | i.v. (tail vein) |

**A**

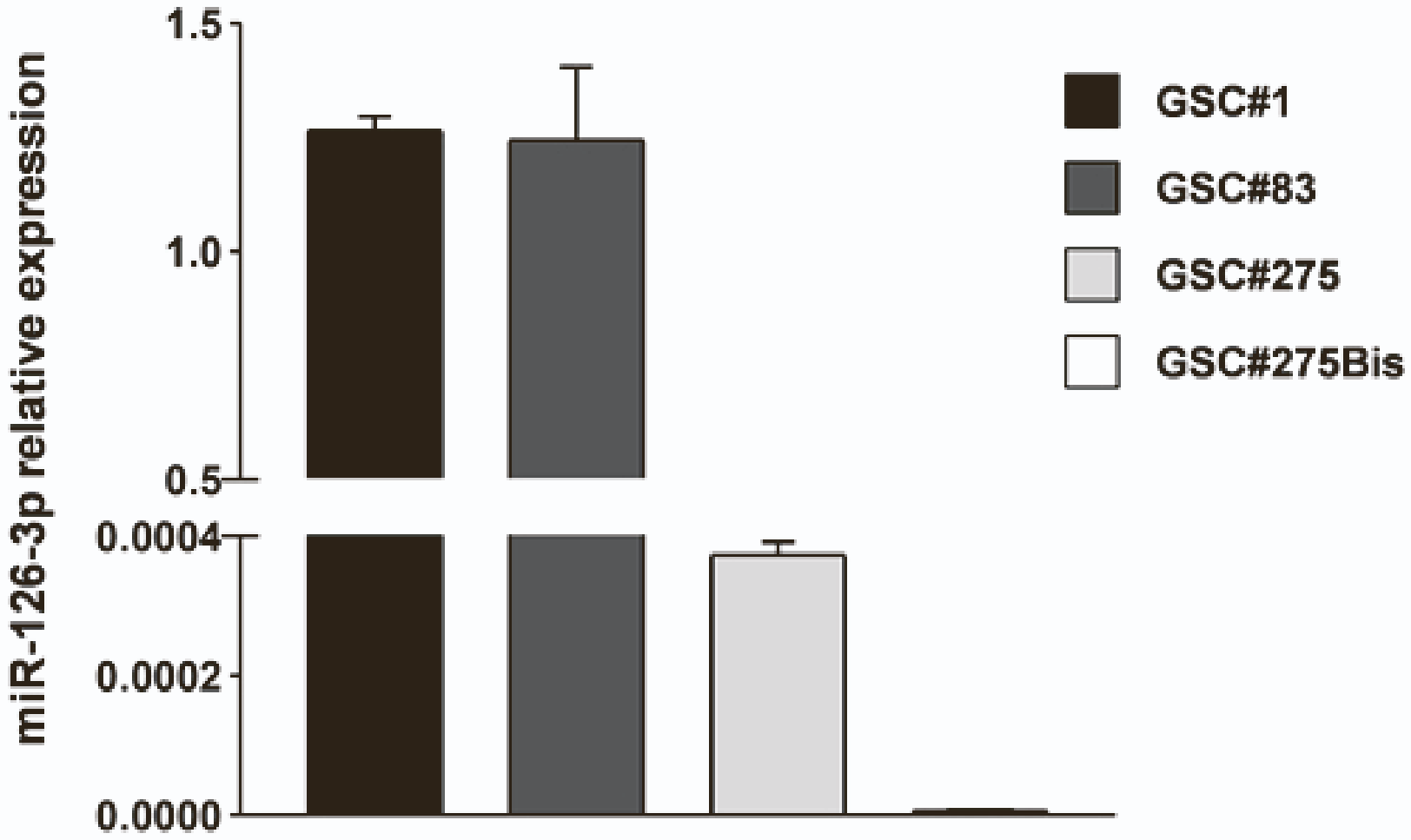


**B**

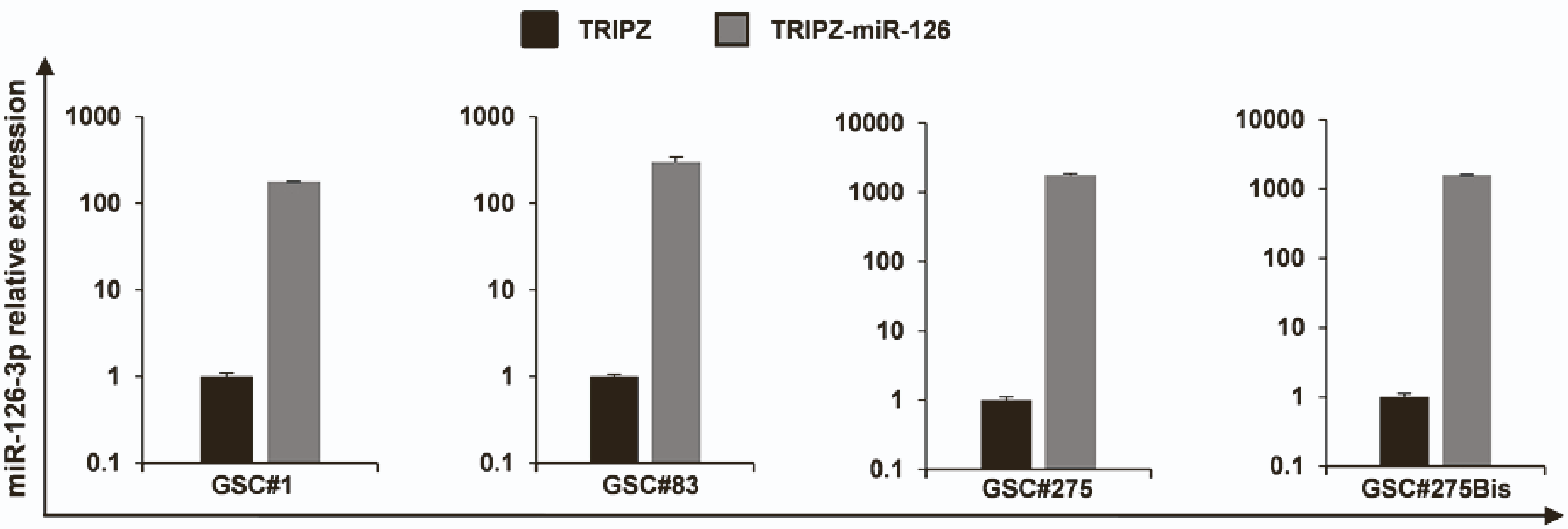


**C**

| | GSC#1 | | GSC#83 | | GSC#275 | | GSC#275Bis | |
|---|---|---|---|---|---|---|---|---|
| | TRIPZ | TRIPZ-miR126 | TRIPZ | TRIPZ-miR126 | TRIPZ | TRIPZ-miR126 | TRIPZ | TRIPZ-miR126 |
| **LAT1** | 27.6 ± 0.1 | 24.4 ± 0.02 | 25.6 ± 0.04 | 27.2 ± 0.1 | 25.8 ± 0.01 | 26.8 ± 0.3 | 25.0 ± 0.07 | 26.1 ± 0.2 |
| **PLXNB2** | 29.5 ± 0.1 | 26.9 ± 0.1 | 34.0 ± 0.2 | 33.9 ± 0.2 | 29.3 ± 0.1 | 30.0 ± 0.03 | 35.0 ± 0.08 | 35.7 ± 0.06 |
| **SPRED1** | 26.7 ± 0.09 | 25.7 ± 0.2 | 26.8 ± 0.06 | 26.6 ± 0.08 | 28.4 ± 0.3 | 28.1 ± 0.2 | 26.9 ± 0.01 | 27.7 ± 0.09 |
| **GAPDH** | 20.9 ± 0.02 | 18.9 ± 0.02 | 22.3 ± 0.2 | 21.6 ± 0.07 | 19.9 ± 0.6 | 19.2 ± 0.6 | 20.8 ± 0.2 | 20.9 ± 0.004 |

**Figure S1. miR-126-3p basal expression on selected GSC lines and effects of miR-126 restoration on its targets. (A)** RT-PCR analysis of miR-126-3p in the 4 selected GSC lines (GSC#1, GSC#83, GSC#275 and GSC#275Bis). **(B)** RT-PCR analysis of miR-126-3p in the 4 selected GSC lines transduced with either TRIPZ or TRIPZ-miR-126 vectors. Samples were run in duplicate. Data were normalized to the RNU6B expression in the corresponding samples. **(C)** Raw Ct values from RT-PCR analysis of LAT1, PLXNB2, and SPRED1 in GSC#1, GSC#83, GSC#275, and GSC#275Bis cells transduced with TRIPZ or TRIPZ-miR-126 vectors. Data are presented as mean ± SD.

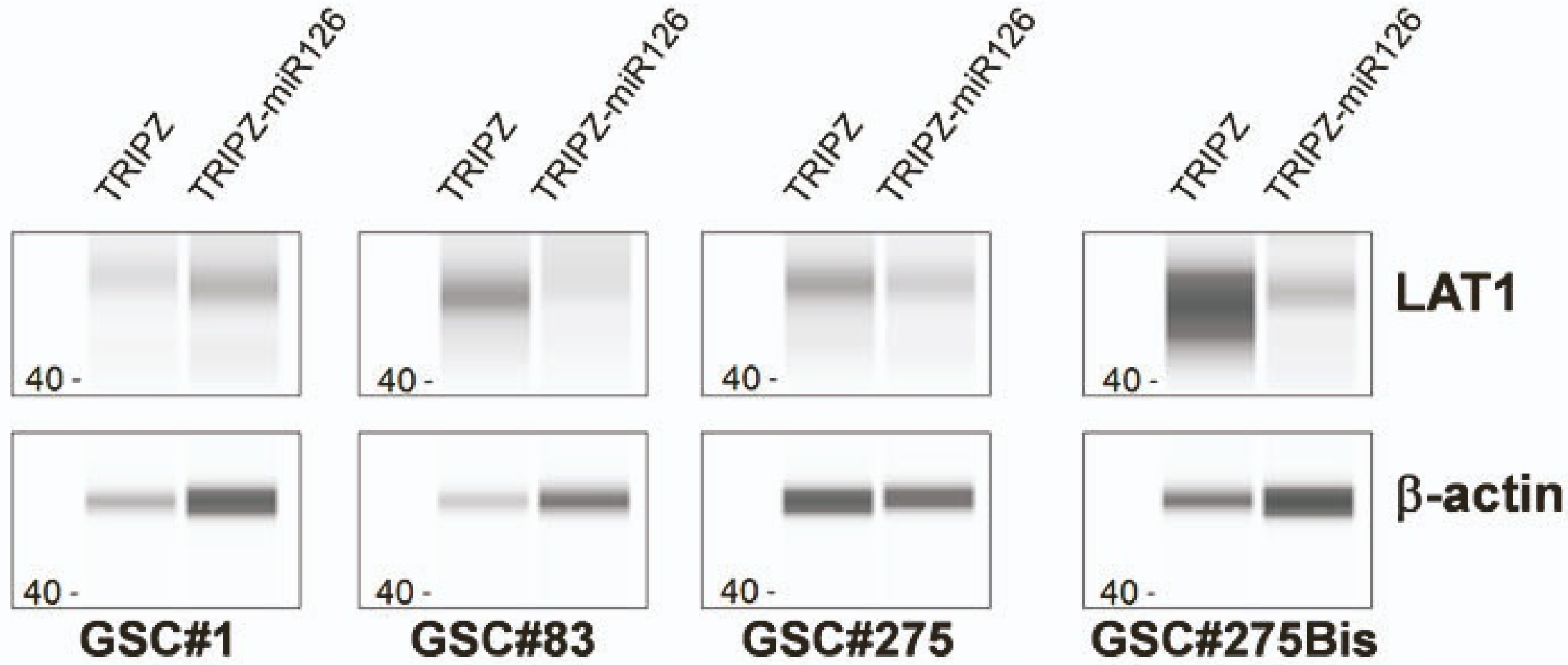


| | TRIPZ | TRIPZ-miR126 | TRIPZ | TRIPZ-miR126 | TRIPZ | TRIPZ-miR126 | TRIPZ | TRIPZ-miR126 |
|---|---|---|---|---|---|---|---|---|
| LAT1 | 1359,1 | 3423,7 | 9855,6 | 1652,8 | 6467,4 | 3049,2 | 31136,2 | 8314,0 |
| β-actin | 3539,7 | 8564,3 | 2521,4 | 5071,3 | 8691,9 | 7165,4 | 5614,1 | 10309,7 |

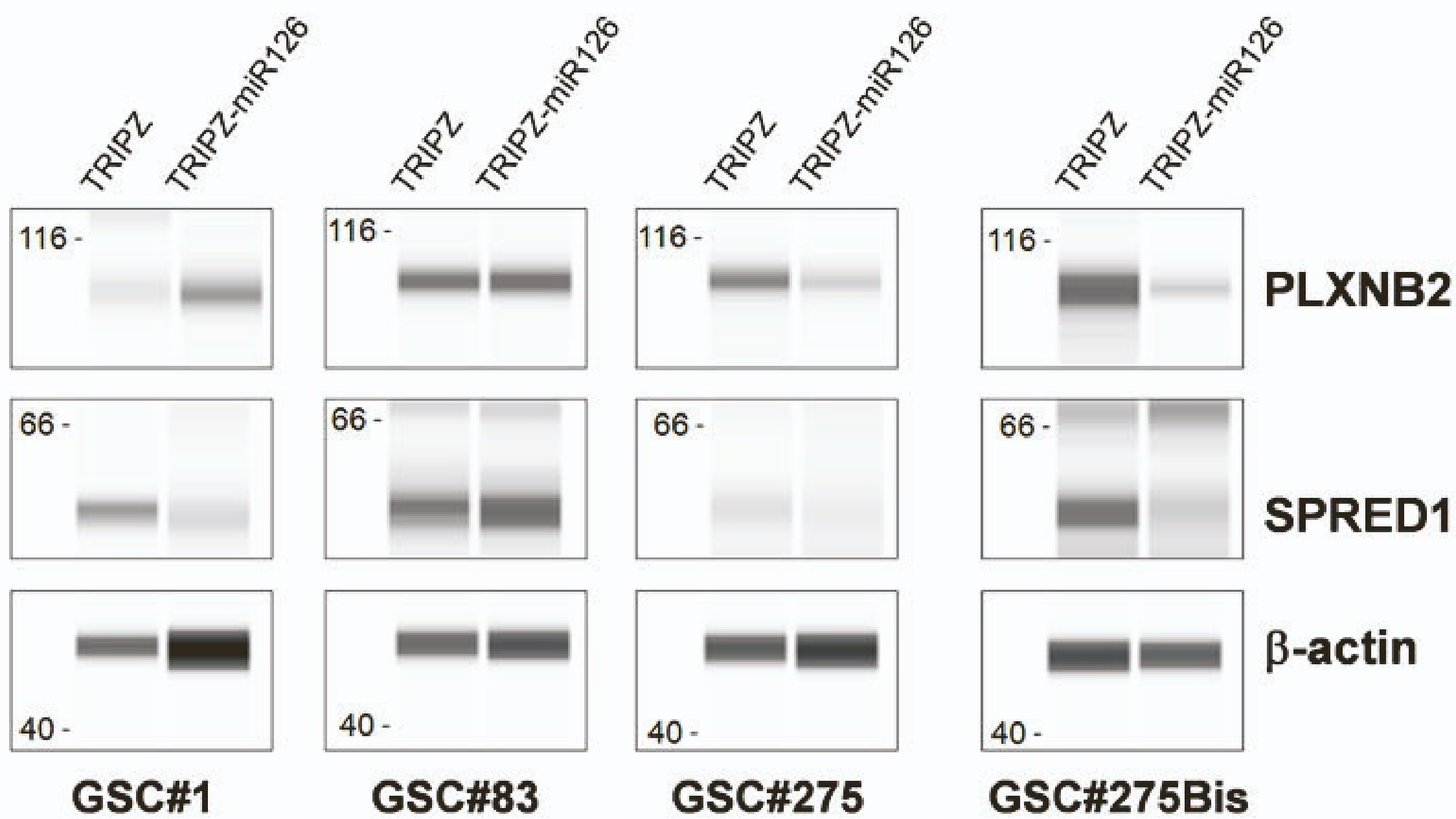


| | TRIPZ | TRIPZ-miR126 | TRIPZ | TRIPZ-miR126 | TRIPZ | TRIPZ-miR126 | TRIPZ | TRIPZ-miR126 |
|---|---|---|---|---|---|---|---|---|
| PLXNB2 | 25317,7 | 40311,9 | 78489,9 | 77913,3 | 42284,9 | 25217,6 | 99457,3 | 33929,8 |
| SPRED1 | 9767,3 | 9822,6 | 22055,3 | 23658,0 | 9618,5 | 8428,4 | 12240,2 | 5456,1 |
| β-actin | 69303,1 | 212044,7 | 157769,3 | 201318,1 | 178311,5 | 261886,2 | 248778,7 | 196959,4 |

**Figure S2. miR-126 restoration induces a significant decrease in the level of target proteins.** WES full-length gel image of LAT1, PLXNB2 and SPRED1 expression in the 4 selected GSC lines (GSC#1, GSC#83, GSC#275 and GSC#275Bis) transduced with either TRIPZ or TRIPZ-miR-126 vectors. Protein expression was quantified using AUC (area under the curve) measurements generated using Compass Software. Signal intensity was normalized to the β-actin expression in the corresponding sample. Representative images from two independent experiments are shown.

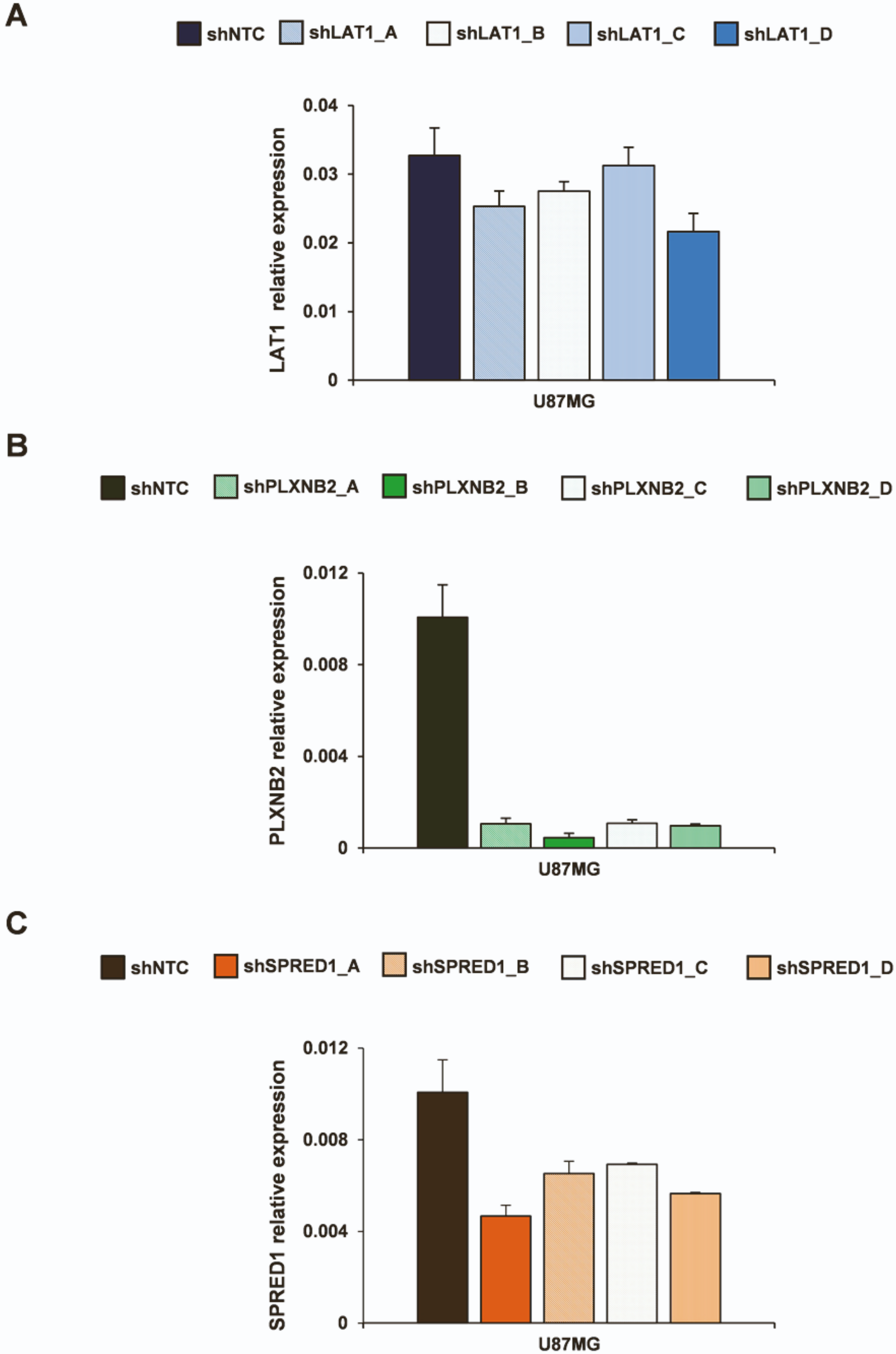


**Figure S3. Selection of the most effective sh-RNA vectors on target silencing in U87MG glioma cell line.** RT-PCR analysis of LAT1 **(A)**, PLXNB2 **(B)**, and SPRED1 **(C)** in U87MG cells transduced with shNTC or four shRNA specific vectors (A–D). Samples were run in duplicate, and data were normalized to GAPDH expression.

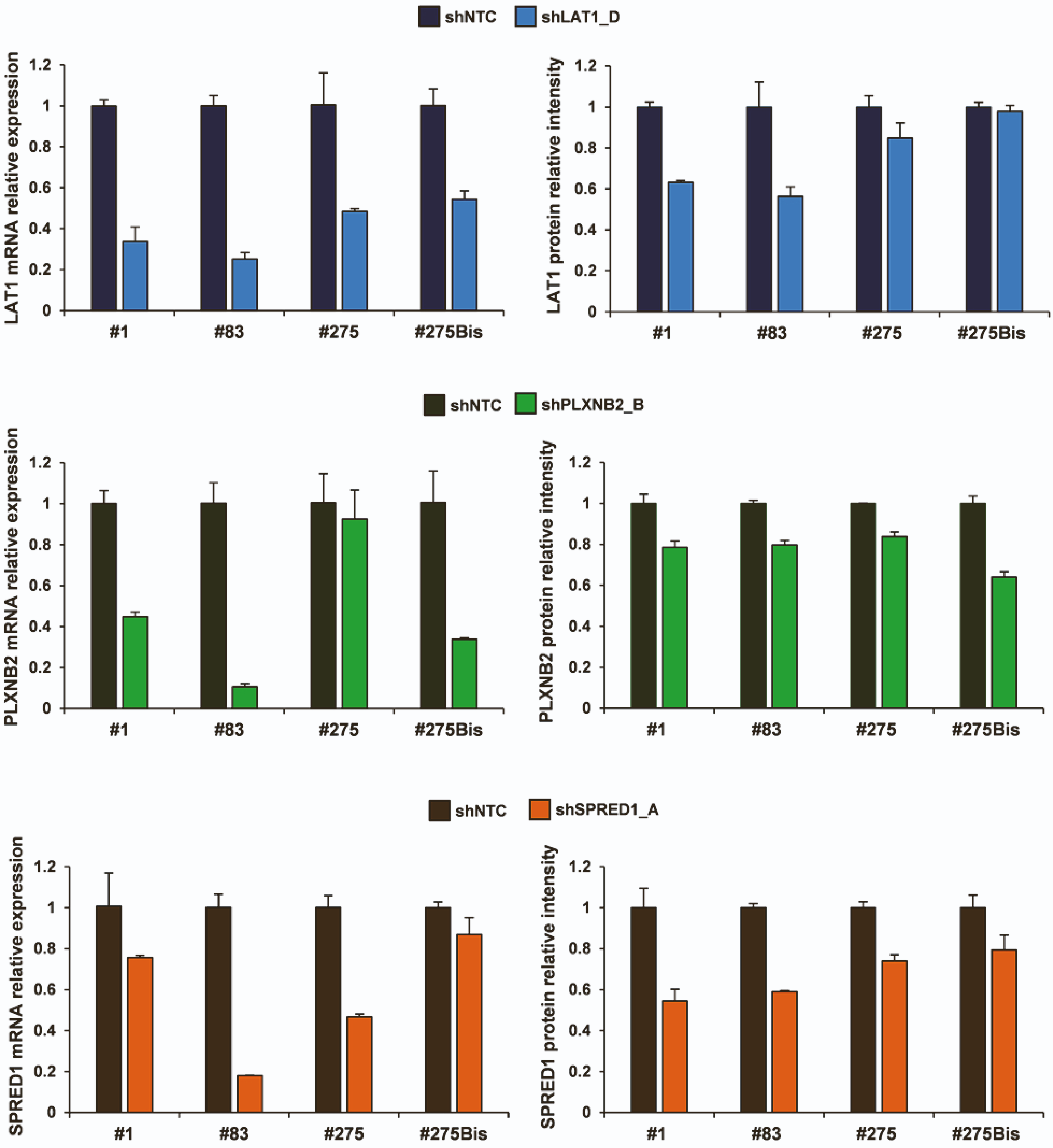


**Figure S4. Effect of the selected shRNA vectors on target silencing.** RT-PCR analysis of LAT1, PLXNB2, and SPRED1 in GSC#1, GSC#83, GSC#275, and GSC#275Bis cells transduced with shNTC or the most effective shRNA vector. Samples were run in duplicate, and data were normalized to GAPDH expression (*left panels*). WES analysis of LAT1, PLXNB2, and SPRED1 in GSC#1, GSC#83, GSC#275, and GSC#275Bis cells transduced with shNTC or the most effective shRNA vector. Protein levels were quantified using AUC measurements and normalized to β-actin expression (*right panels*).

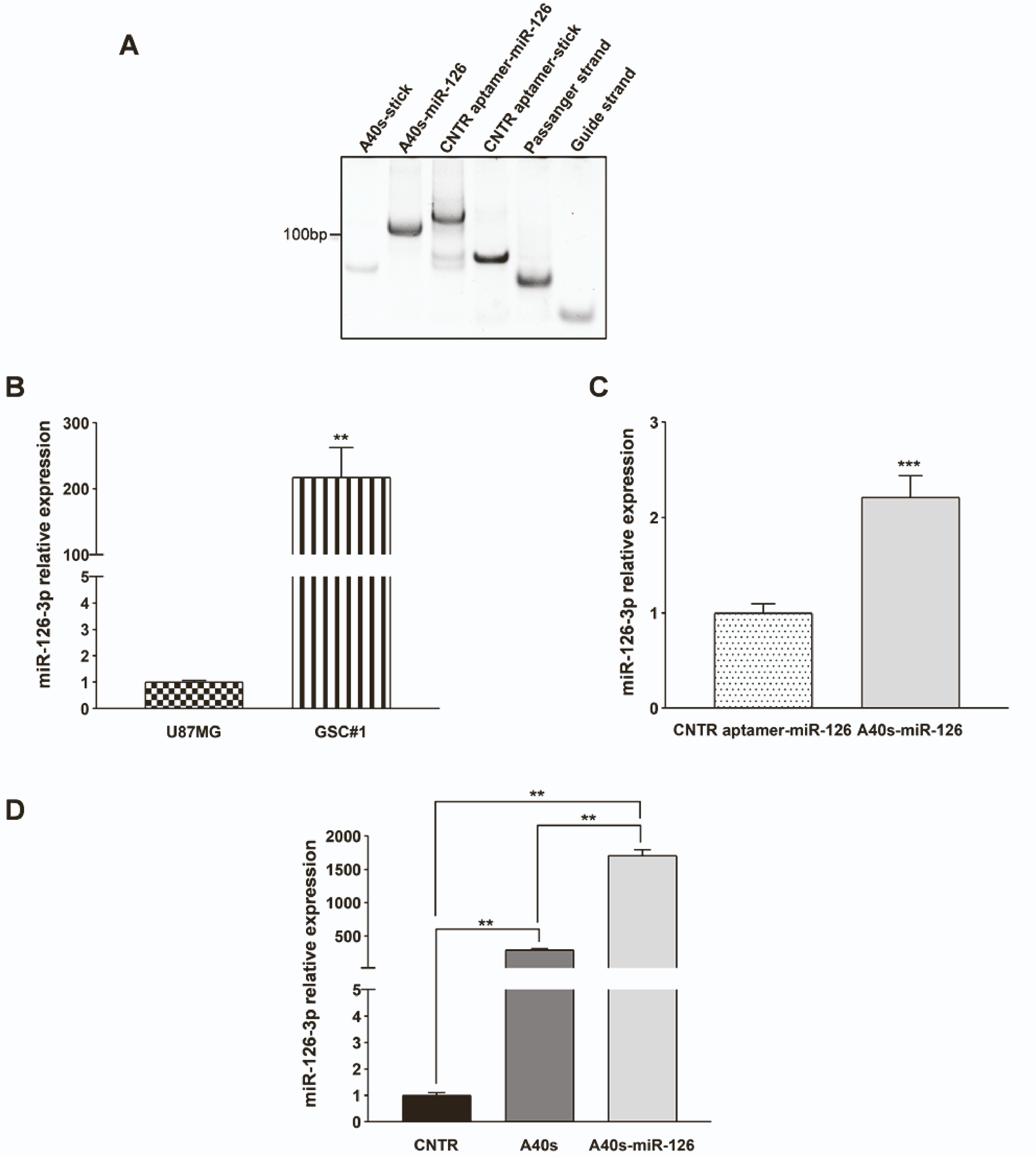


**Figure S5. Successfully annealing of the A40s-miR-126-3p chimera. (A)** Non-denaturing gel electrophoresis analysis reporting correct annealing of the A40s-miR126-3p chimera conjugate. **(B)** Relative miR-126-3p levels assessed by RT-PCR after incubation of GSC#1 with A40s-miR126 chimera and compared to U87MG as an EphA2 negative cell line. Bars report mean of three independent experiments ± SD. **p ≤ 0.01(Student's t test). **(C)** Relative miR-126-3p levels assessed by RT-PCR after incubation of GSC#1 with A40s-miR126 chimera and compared with an unrelated sequence-based chimera as a control. Bars report mean of three independent experiments ± SD. ***p ≤ 0.001 (Student's t test). **(D)** RT-PCR for miR-126-3p expression of no treated GSC#1 (CNTR), GSC#1 incubated with A40s-aptamer alone (A40s) or A40s-miR126-3p chimera (A40s-miR126).

**A**

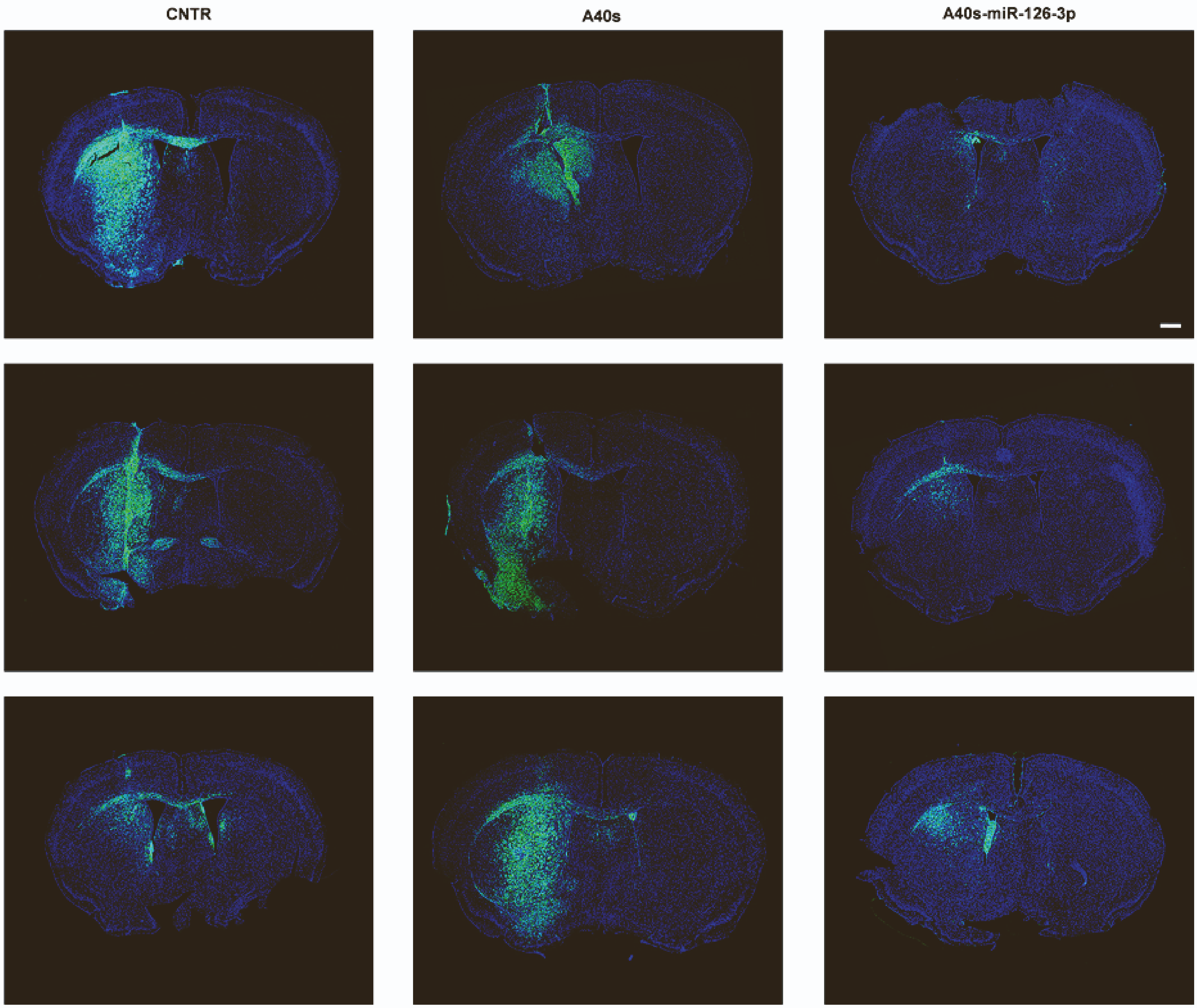


**B**

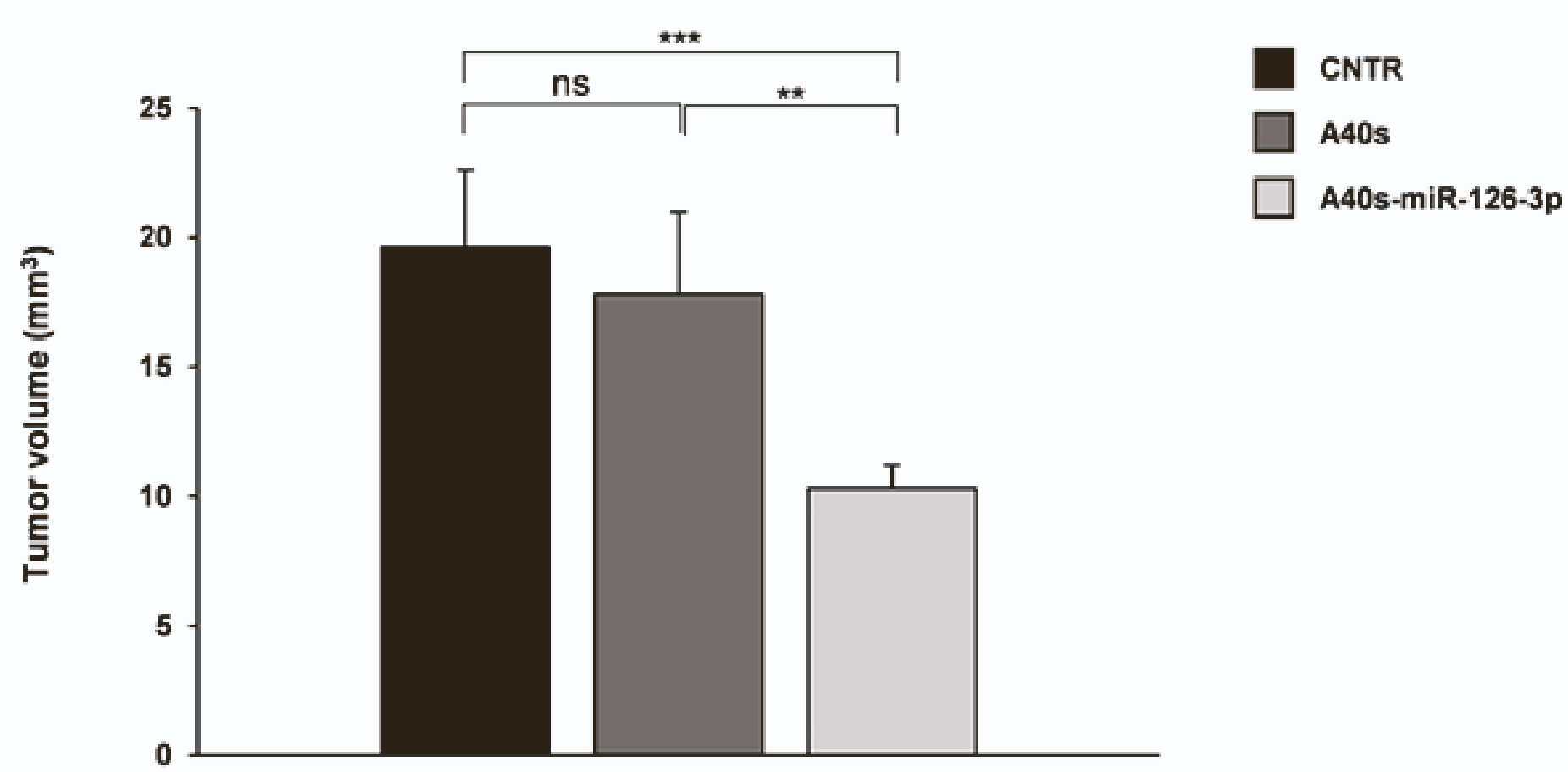


**Figure S6 The in vivo effects of aptamer A40s-miR-126-3p on tumor xenografts in mice.** **(A)** Images of coronal mouse brain sections at 8 weeks after the intracranial injection of GFP$^+$ GSC#1. One week after grafting mice were randomly assigned to three groups: saline treated (CNTR), treated with A40s-aptamer alone (A40s) or A40s-miR-126-3p chimera (A40s-miR-126-3p). Free floating brain slices were counterstained with DAPI (blue). Scale bar: 500 μm. **B** Graph showing the tumor volume for the intracranial tumors after 8 weeks from the injection of GSCs. ns (not significant), **p<0.01, ***p<0.001 based on Student's t test
