# Supplementary material for: Aptamer-guided delivery of miR-126 suppresses glioblastoma stem-like cell invasion and tumor progression
Source: Mol Ther Nucleic Acids. 2026 Sep 17;37(4):103103. doi: 10.1016/j.omtn.2026.103103 (PMC13639097; doi:10.1016/j.omtn.2026.103103)
Supplement: Supplementary file 2 [file mmc2.pdf]

Molecular Therapy
Nucleic Acids
Original Article

Aptamer-guided delivery of miR-126 suppresses glioblastoma stem-like cell invasion and tumor progression

Giorgia Castellani,[1,13] Mariachiara Buccarelli,[1,13] Alessandra Affinito,[2] Gabriele De Luca,[1] Ramona Ilari,[1] Francesca Pedini,[1] Alessandra Di Giamberardino,[1] Mariagiovanna Ballato,[3] Gabriele Ricciardi,[3] Martina Offi,[4,5] Cristiana Mollinari,[6,7] Nadia Felli,[1] Alessandra Boe,[8] Giovanna Marziali,[1] Mauro Biffoni,[1] Maria Caffo,[9] Gerolama Condorelli,[10,11] Maurizio Martini,[12] Quintino Giorgio D'Alessandris,[5,14] and Lucia Ricci-Vitiani[1,14]

[1]Department of Oncology and Molecular Medicine, Istituto Superiore di Sanità, 00161 Rome, Italy; [2]IRCCS SYNLAB SDN, 80143 Naples, Italy; [3]Department of Biomedical, Dental, Morphological and Functional Imaging Sciences, University of Messina, 98122 Messina, Italy; [4]Department of Neuroscience, Catholic University School of Medicine, 00168 Rome, Italy; [5]Department of Neurosurgery, Fondazione Policlinico Universitario A. Gemelli IRCCS, 00168 Rome, Italy; [6]Department of Neuroscience, Istituto Superiore di Sanità, 00161 Rome, Italy; [7]Institute of Translational Pharmacology, National Research Council, 00133 Rome, Italy; [8]Core Facilities, Istituto Superiore di Sanità, 00161 Rome, Italy; [9]Biomedical and Dental Sciences and Morphofunctional Imaging, Unit of Neurosurgery, University of Messina, 98122 Messina, Italy; [10]Department of Molecular Medicine and Medical Biotechnology, University of Naples Federico II, 80131 Naples, Italy; [11]Institute of Endotypes in Oncology, Metabolism and Immunology "G. Salvatore" (IEOMI), Consiglio Nazionale Delle Ricerche (CNR), 80131 Naples, Italy; [12]Department of Human Pathology in Adult and Developmental Age "Gaetano Barresi", University of Messina, 98122 Messina, Italy

Glioblastoma, IDH-wildtype (GBM) is the most common and aggressive primary brain tumor in adults, with a median overall survival of 15–18 months. New therapeutic targets are needed to improve patients' prognosis. Due to their powerful regulatory capabilities, miRNAs could be possible candidate targets. MiR-126 is considered a tumor suppressor in several cancers, including GBM. In a previous study, we found that the infiltrative shift observed in response to bevacizumab in a GBM mouse model, induced a high miR-126 downregulation, confirming its tumor-suppressor function, exerted by inhibiting growth and angiogenesis. Here, we either stably restored miR-126 expression in GBM stem-like cells by transduction or transiently delivered it through an A40s aptamer-based conjugate. Both interventions led to an overall impairment of tumorigenic properties. Notably, *in vivo* systemic treatment with an aptamer-miR-126 chimera significantly reduced brain infiltration in GBM mouse models. Among miR-126 potential targets, we confirmed the essential amino acid transporter LAT1, the transmembrane receptors PLXNB2 and SPRED1 as responsible for miR-126 negative regulation of GBM development. These findings corroborate miR-126 as both a putative prognostic biomarker and a functional tumor-suppressor in GBM, identifying the aptamer-miR126 chimera as a novel potential candidate for a targeted therapeutic approach for more effective GBM management.

INTRODUCTION

Glioblastoma, IDH-wildtype (GBM) represents the most lethal primary brain malignancy in adults and remains one of the most therapeutically challenging solid tumors. GBM is characterized by profound molecular heterogeneity, aggressive infiltrative growth, marked angiogenesis, and a highly resilient subpopulation of glioblastoma stem-like cells (GSCs).[1,2] Despite significant technological advances in neurosurgery, neuroimaging, radiation, and chemotherapy protocols, median overall survival has stagnated around 15–18 months, and more than 90% of patients succumb to the disease within three years after diagnosis.[3] This disappointing clinical scenario underscores the urgent need for molecularly precise therapeutic strategies capable of targeting the biological processes that sustain tumor growth and recurrence.

Among the regulatory determinants altered in GBM, microRNAs (miRNAs) have emerged as crucial modulators of tumor behavior. These small non-coding RNAs (ncRNAs) orchestrate broad gene networks involved in proliferation, survival, metabolic remodeling, angiogenesis, and stemness.[4] Alteration of miRNA expression is a hallmark of brain cancers, and many miRNAs exert context-specific oncogenic or tumor suppressor roles depending on cellular environment and target availability.[5]

Received 22 May 2026; accepted 15 September 2026;
https://doi.org/10.1016/j.omtn.2026.103103.

[13]These authors contributed equally

[14]Senior author

Correspondence: Quintino Giorgio D'Alessandris, Department of Neurosurgery, Fondazione Policlinico Universitario A. Gemelli IRCCS, 00168 Rome, Italy..
E-mail: quintinogiorgio.dalessandris@policlinicogemelli.it
Correspondence: Lucia Ricci-Vitiani, Department of Oncology and Molecular Medicine, Istituto Superiore di Sanità, 00161 Rome, Italy.
E-mail: lucia.riccivitiani@iss.it

© 2026 The Authors.
Published by Elsevier Inc. on behalf of The American Society of Gene and Cell Therapy.
This is an open access article under the CC BY license (http://creativecommons.org/licenses/by/4.0/).

Within this complex framework, miR-126 has gained increasing attention due to its pleiotropic involvement in endothelial cell biology, vascular integrity, and neoplastic progression.

MiR-126 is encoded within intron 7 of the *EGFL7* gene on chromosome 9q34.3 and is processed into two mature forms, miR-126-3p and miR-126-5p. Associated with endothelial function, *EGFL7*/miR-126 co-transcription regulates vascular stability and modulates vascular endothelial growth factor (VEGF)-dependent angiogenic signaling. Although early studies proposed a potential oncogenic role for miR-126 in specific hematologic malignancies, strong evidence now supports its predominant tumor-suppressor activity across multiple solid tumors, including lung,[6] breast,[7] ovarian,[8] colorectal,[9] and pancreatic[10] cancers. In many of these contexts, miR-126 restrains tumor growth by repressing pathways that promote cell migration, invasion, epithelial-to-mesenchymal transition (EMT), and angiogenesis.[11]

MiR-126 plays a dual role in cancer progression by promoting angiogenesis and maintaining vascular stability, as well as contributing to cancer cell growth. Its expression is elevated in endothelial progenitor cells (EPCs), where miR-126 promotes the proliferation, invasion, and migration of EPCs, while inhibiting apoptosis, through the regulation of Notch1 pathway.[12]

A pilot translational study in GBM revealed that miR-126 is significantly downregulated in the tumor tissue relative to the adjacent non-neoplastic brain parenchyma, and importantly patients with relatively preserved intratumoral (i.t.) miR-126 expression display longer survival than those exhibiting deeper suppression.[13] Another study demonstrated that epigenetic modification is a crucial mechanism in the control of miR-126 expression in glioma, showing that the extent of methylation is associated with tumor grade and confirming that reduced expression of miR-126 leads to gliomagenesis and progression.[14] These findings position miR-126 as both a putative prognostic biomarker and a functional tumor-suppressor miRNA in GBM biology.

In this study, we confirm a critical role for miR-126 in the regulation of GBM development exerting its anti-tumor effects by repressing key oncogenic pathways. It not only negatively regulates pro-angiogenic mediators such as VEGF-A but also impacts cellular proliferation, migration, and EMT through repression of targets including SLC7A5/LAT1, PLXNB2, and SPRED1, all of which have been implicated in GBM aggressiveness and metabolic rewiring.[15–17] Restoration of miR-126 expression in patient-derived GSCs results in marked reductions in proliferation, clonogenicity, migration, and angiogenesis, consistent with a broad repression of tumor-promoting programs. These effects reflect the centrality of miR-126 in controlling transcriptional and metabolic networks that confer resilience to the stem-like tumor compartment.

For a potential therapeutic relevance, the primary obstacle to miR-126-based therapy is an efficient and selective delivery into GBM cells, particularly GSCs located within invasive niches and protected by the blood-brain barrier (BBB). In recent years, however, nucleic acid aptamers have emerged as highly attractive vehicles for targeted delivery.[18] Aptamers are short single-stranded oligonucleotides capable of folding into complex three-dimensional structures with affinity and specificity similar to antibodies. They exhibit low immunogenicity, rapid tissue penetration, chemical versatility, and, in many cases, the capacity to cross the BBB. Their ability to internalize into target-expressing cells further enhances their appeal as molecular carriers for therapeutic oligonucleotides.

Among aptamers developed against GBM, those targeting GSC-specific markers represent the most promising therapeutic tools. Through a rigorous cell-SELEX process performed on multiple patient-derived GSC lines, the RNA aptamer A40s was identified as a high-affinity ligand selectively binding GSCs while sparing differentiated GBM cells.[19] A40s recognizes ephrin type-A receptor 2 (EphA2), a tyrosine kinase receptor crucial for GSC self-renewal, tumorigenicity, and therapeutic resistance.[20] Extensive *in vitro* studies confirmed that A40s internalizes rapidly into GSCs and selectively stains stem-like cells in patient tissues, thereby validating its potential as both a diagnostic probe and a therapeutic delivery system.

RESULTS

MiR-126 expression in GBM tissues and GSCs correlates with GBM patient survival

The role of miR-126 in GBM carcinogenesis remains controversial as to whether miR-126 is a tumor-suppressive or oncogenic miRNA. A study comparing the expression of miR-126 in oligodendroglioma and astrocytoma found that astrocytomas expressed less miR-126 than oligodendrogliomas.[21] In another study comparing miRNA profiles of GBM spheroid cultures (GSCs) to GSC-derived orthotopic xenografts, miR-126 resulted one of the three most overexpressed miRNAs in the non-differentiated GSCs.[22]

To verify miR-126 expression in our cohort of patients, we analyzed by real-time PCR, miR-126-3p expression levels in 58 human GBM tissues and in 56 patient-derived GSC lines. The main clinicopathological characteristics of the 58 patients whose tumor samples were included in the GBM tissue cohort for the survival analysis are summarized in Table S1.

Interestingly, Kaplan-Meier analyses showed that low miR-126-3p expression was significantly associated with shorter overall survival both in the GBM tissue cohort (n = 58; p = 0.0438, HR = 2.26, 95% confidence interval [CI]: 1.023–4.993) and among patients from whom the GSC cultures were established (n = 56; p = 0.0149, HR = 2.295, 95% CI: 1.176–4.479) (Figures 1A and 1B). These data support a role for miR-126 as both a putative prognostic biomarker and a functional tumor-suppressor miRNA in GBM.

In vitro restoration of miR-126 impairs tumorigenic properties of GSCs

To investigate the role of miR-126 on GSC tumorigenic properties, we overexpressed miR-126 in patient-derived GSC lines by using

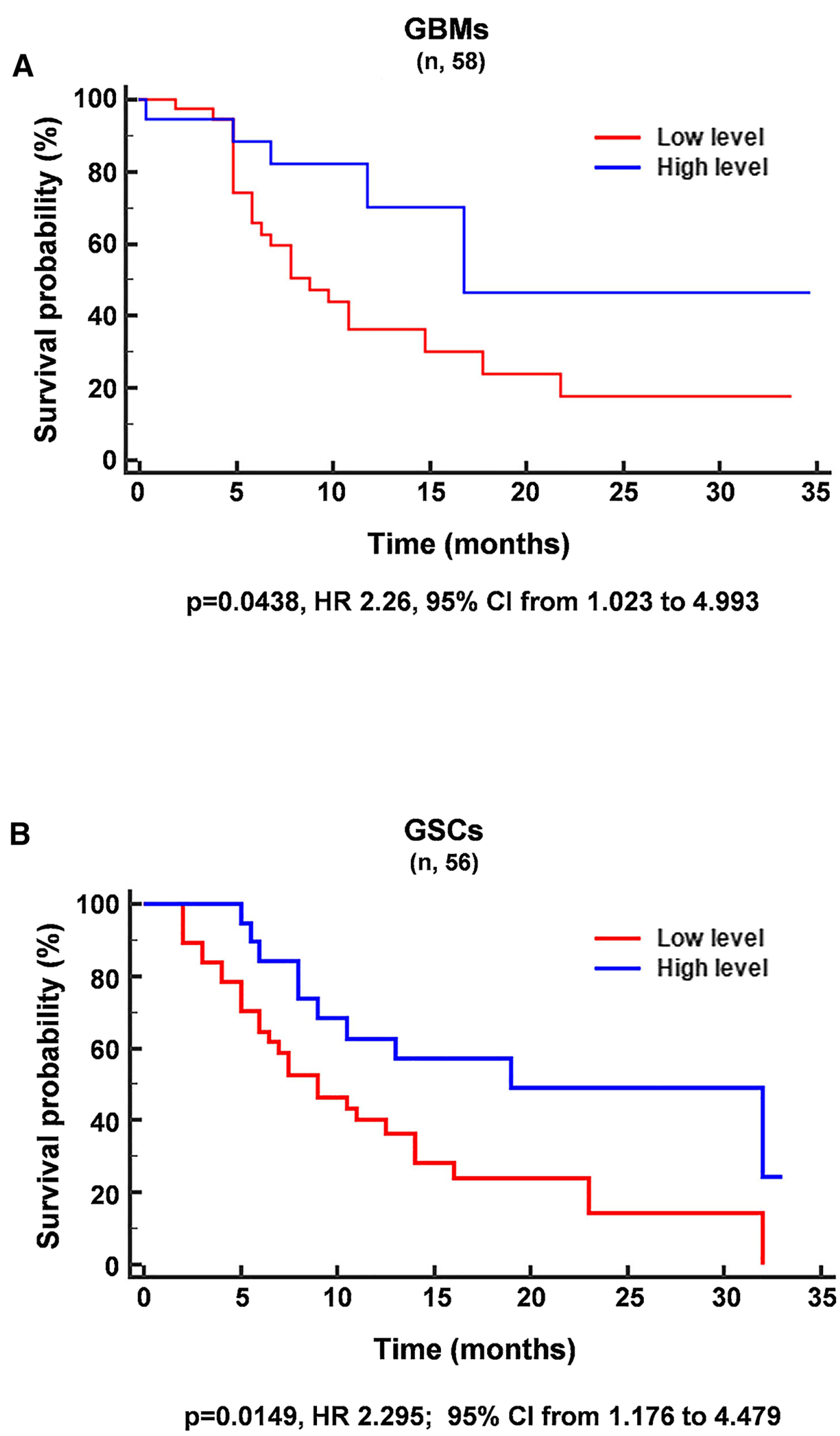

Figure 1. Association between miR-126-3p expression and overall survival in GBM

Kaplan-Meier survival analyses of patients stratified according to miR-126-3p expression in GBM tissues (A; n = 58) and patient-derived GSC cultures (B; n = 56). Within each cohort, the mean miR-126-3p expression value was used as the cutoff to define high- and low-expression groups. Approximately 30% of the GSC cultures were derived from tumors also included in the GBM tissue cohort. Low miR-126-3p expression was significantly associated with shorter overall survival in both cohorts. Hazard ratios, 95% confidence intervals, and log rank p values are reported.

an inducible Tet-On lentiviral vector (TRIPZ) carrying pri-miR-126 in the 3′ untranslated region of Red Fluorescent Protein (RFP).[23] Two GSC lines (i.e., GSC#1 and GSC #83) and one pair of GSC lines derived from the same patient at first surgery (GSC#275) and at surgery for recurrence (GSC#275Bis), were chosen as representatives of independent patient-derived GSC cultures with stable stem-cell characteristics.[24] Basal miR-126-3p expression varied among the four patient-derived GSC lines, as shown in Figure S1A. These cell lines were transduced and exposed to doxycycline, RFP-positive cells were flow-sorted and miR-126 restoration was confirmed by real-time PCR (Figure S1B).

Lentiviral transduction efficiently restored miR-126 expression in all cell lines, compared to empty vector (TRIPZ)-transduced cells, although the magnitude of miR-126 restoration differed among GSC lines, reflecting intrinsic biological variability and different endogenous miR-126-3p basal expression levels (Figures S1A and S1B). Ectopic expression of miR-126 significantly impaired cell growth of all the GSC lines tested (Figures 2A–2D), with a stable decrease in growth rate. Importantly, the biological effects of miR-126 restoration were reproducible despite the heterogeneous basal expression observed across independent patient-derived GSC cultures, supporting the robustness and generalizability of the observed phenotype.

We then examined whether miR-126 could alter additional malignant features of GSCs, such as colony formation and migration abilities. After miR-126 induction, TRIPZ-miR-126 GSCs formed significantly fewer colonies than TRIPZ cells in all GSC lines except for GSC#275 (Figures 2A–2D).

Furthermore, the motility of transduced GSCs was examined and a significant reduction in the migration capabilities of TRIPZ-miR-126 GSCs, compared to TRIPZ-transduced GSCs, was observed in GSC#1 and #275Bis lines (Figures 2A and 2D). We also observed a decrease, although not significant, in migratory capacity in GSC#83 cells (Figure 2B) while, in GSC#275 cells, the induction of miR-126 did not influence cell motility (Figure 2C). Thus, miR-126 restoration resulted in a considerable inhibition of cell proliferation of all GSCs tested, while inhibition of migration and colony formation was not observed in all GSC lines tested, suggesting that, although miR-126 is a tumor suppressor in GBM, it acts by interfering with different molecular pathways depending on the genetic background of the patient from which the GSC line was derived.

Tumor suppressor function of miR-126 involves GSC interaction with tumor microenvironment

To further investigate the regulatory network of miR-126 and identify the key mediators of its tumor suppression function, we screened a list of its potential target mRNAs obtained using TargetScan 7.0 and MicroRNA Target Prediction Database (miRDB) algorithms. Further selection was performed by matching these miR-126 potential targets with those validated in literature, based on their relationship with GBM. As validated miR-126 targets, we selected L-type amino acid transporter, (LAT1/SLC7A5), Plexin B2, (PLXNB2), and Sprouty-related EVH1 domain containing 1, (SPRED1).[25–27] All these genes have been implicated in the pathogenesis of GBM. Particularly, LAT1 has a clear role in mediating cell growth and proliferation in different tumors, including GBM[15]; PLXNB2 has a primary role in promoting tumor infiltration[28]; SPRED1 has been studied in several pathological conditions, including cancer, due to its Sprouty-related function in angiogenesis.[29]

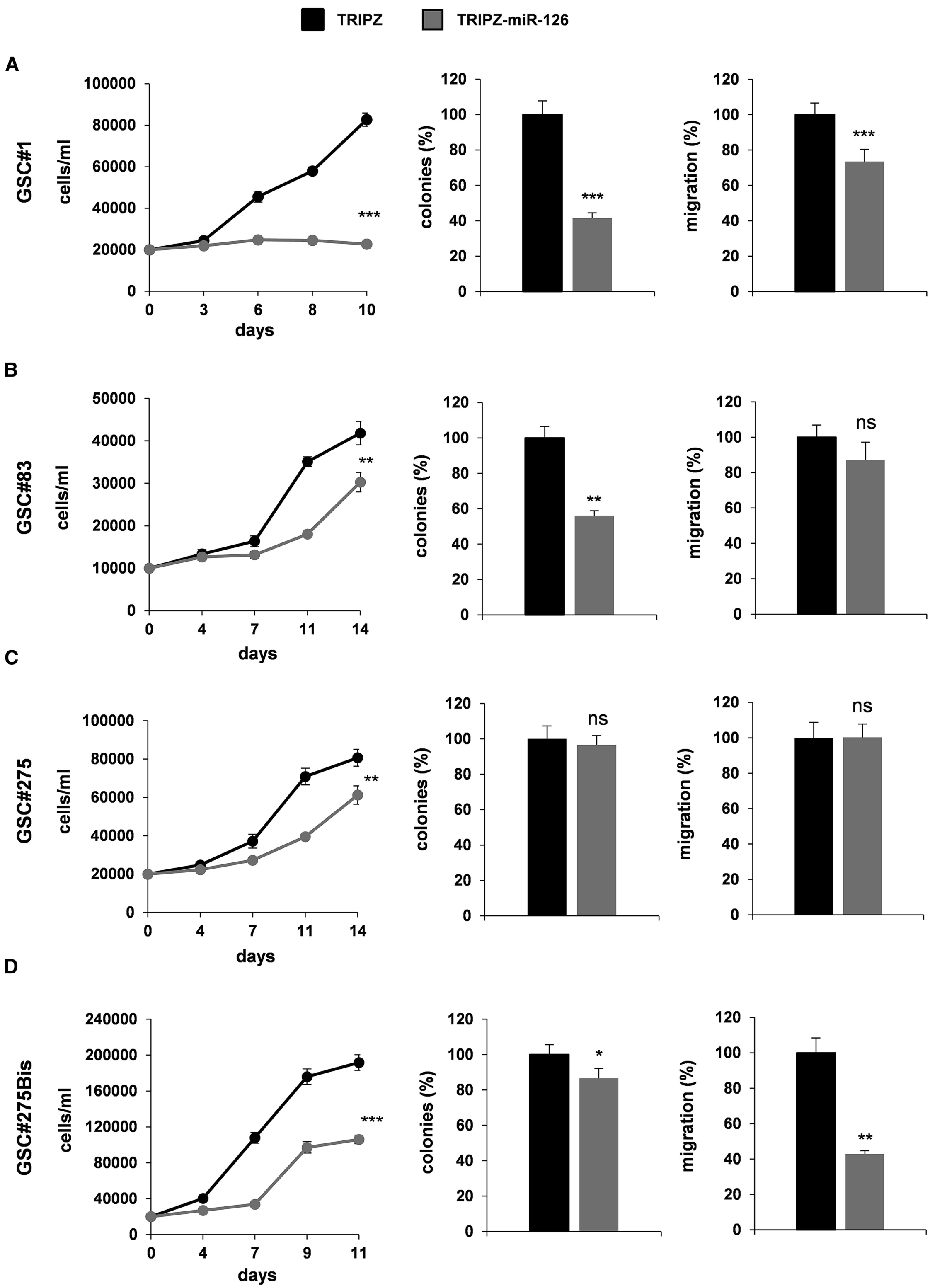

(legend on next page)

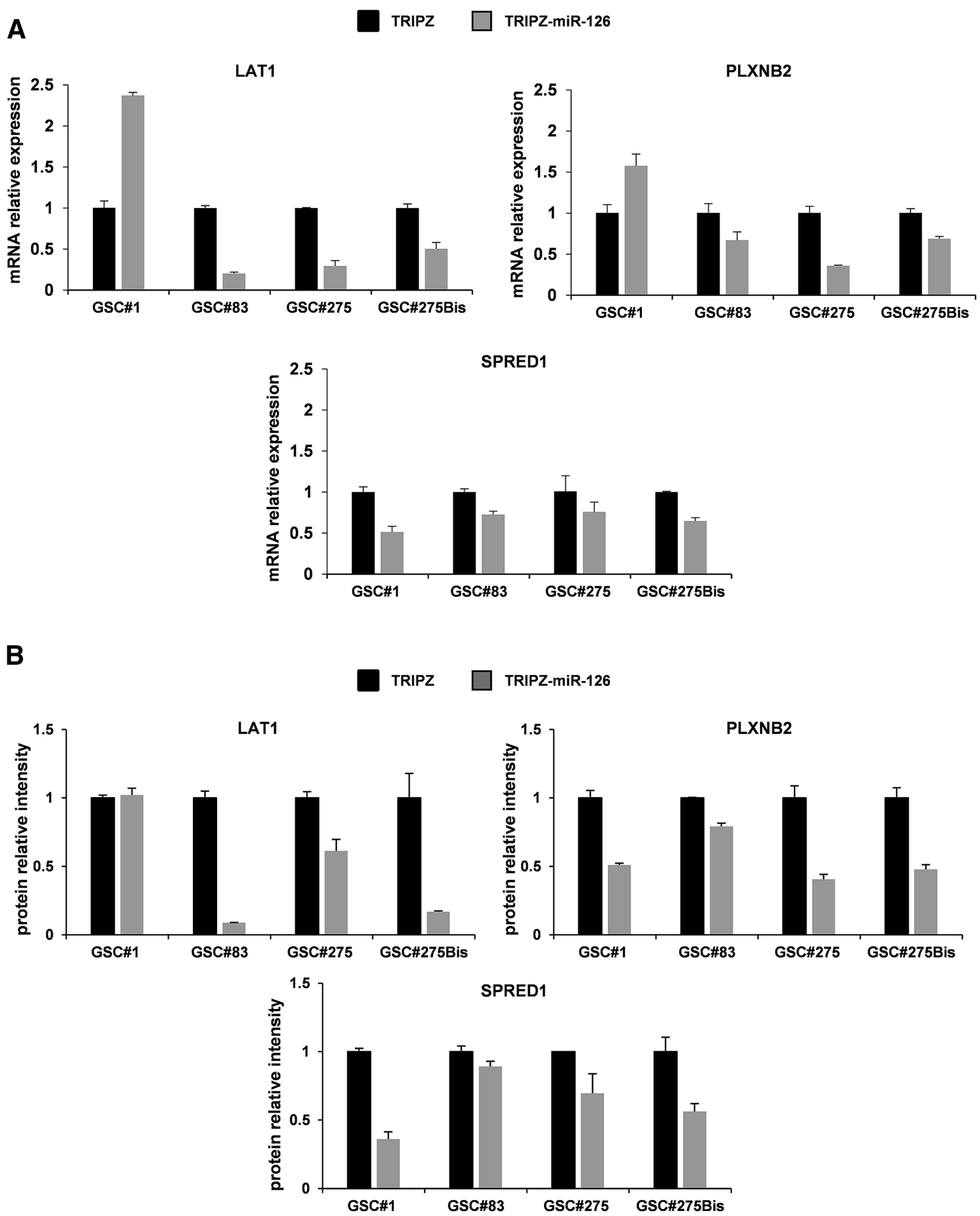

Figure 3. miR-126 overexpression induces a significant decrease in the level of target proteins
(A) Real-time PCR analysis of LAT1, PLXNB2, and SPRED1 expression in GSC#1, GSC#83, GSC#275, and GSC#275Bis transduced with TRIPZ or TRIPZ-miR-126 vectors. Samples were run in duplicate. Data were normalized to the GAPDH expression in the corresponding samples. (B) WES analysis of LAT1, PLXNB2, and SPRED1 expression in GSC#1, GSC#83, GSC#275, and GSC#275Bis transduced with TRIPZ or TRIPZ-miR-126 vectors. The protein expression was quantified using AUC (area under the curve) measurements generated using Compass Software. Signal intensity was normalized to the β-actin expression in the same samples.

To investigate whether miR-126 could interfere with the tumorigenic properties of GSCs by targeting LAT1, SPRED1, and PLXNB2, we evaluated the expression of these potential targets in GSCs transduced with TRIPZ-miR-126 compared to those transduced with empty vector (TRIPZ), at the mRNA and protein levels (Figures 3A and 3B). Restoration of miR-126 differentially affected the expression of its predicted targets across the four patient-derived GSC lines (Figures 3A, 3B, and S1C). Following miR-126-3p restoration, target modulation was heterogeneous at the transcript level and did not invariably parallel changes in protein abundance. Nevertheless, protein analysis confirmed modulation of the miR-126-3p target network across the four GSC lines (Figures 3B and S2). As shown in Figures 3A and 3B, except for LAT1 and PLXNB2 in GSC#1, miR-126 restoration reduced the expression of the three targets at both the mRNA and protein levels in all tested GSC lines. The reduction was particularly remarkable at the protein level (Figures 3B and S2); in detail, LAT1 protein expression was reduced by more than 50% in GSC#83 and #275Bis whereas, PLXNB2 and SPRED1 protein expression was reduced in all the GSC lines tested even though to a different extent. These differences confirm that LAT1, PLXNB2, and SPRED1 are miR-126-3p targets in GSCs, as well as, the heterogeneity of the molecular mechanisms underlying its tumor suppressor function.

The partial discordance observed between transcript and protein levels is compatible with the multiple mechanisms through which miRNAs regulate gene expression, including both mRNA destabilization and translational repression, although additional post-transcriptional regulatory mechanisms cannot be excluded.[30]

To dissect the molecular mechanisms underlying the tumor suppressor function of miR-126 and to evaluate the relative contribution of its targets, we examined the effect of target silencing by transducing

Figure 2. miR-126 overexpression reduces tumorigenic abilities of GSCs
Functional *in vitro* assays on (A) GSC#1, (B) GSC#83, (C) GSC#275, and (D) GSC#275Bis transduced with either TRIPZ or TRIPZ-miR-126 vectors. Growth curves of GSC lines transduced with TRIPZ or TRIPZ-miR-126 vectors (left). Points and range lines at each day represent mean ± SD of at least two independent experiments in triplicate. Colony formation assay of GSC lines transduced with TRIPZ or TRIPZ-miR-126 vectors (middle). Percent colony number values from two independent experiments in triplicate were calculated over the correspondent control vector and are shown as mean ± SD for each GSC line. Migration assay of GSC lines transduced with TRIPZ or TRIPZ-miR-126 vectors (right). Values are reported as percentage relative to control vector and shown as mean ± SD from two independent experiments in triplicate. ns, not significant; *$p < 0.05$; **$p < 0.01$; ***$p < 0.001$ based on Student's t test.

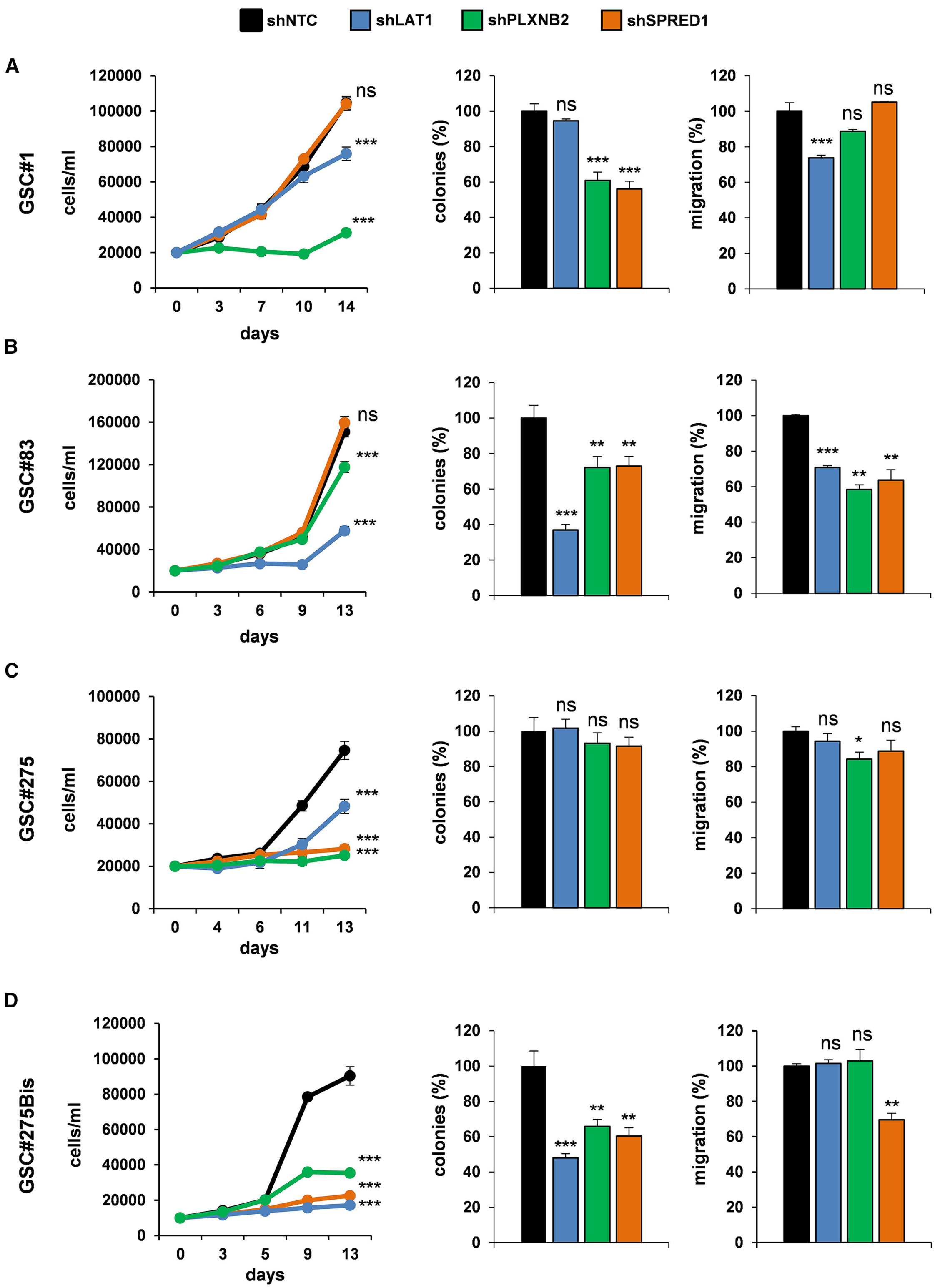

(legend on next page)

lentiviral vector carrying shLAT1, shPLXNB2, shSPRED1, or shNTC (short hairpin no target control) sequences and green fluorescent protein (GFP) as a reporter in the four selected GSC lines (i.e., GSC#1, #83, #275, and #275Bis). For each gene, we selected by real-time PCR the construct, out of four, able to better silence the target gene (Figure S3). Using the most effective construct, we then evaluated the reduction in endogenous mRNA and protein target levels in the four GSC lines tested. Again, we observed different levels of silencing in the four GSC lines suggesting different basal expression levels of the corresponding gene (Figure S4). We then evaluated the effect of silencing on proliferation, clonogenic and migration ability, *in vitro* (Figures 4A–4D). Surprisingly, LAT1 knockdown induced a significant decrease of cell proliferation (Figures 4A–4D, left) even in GSC#275Bis, where we observed only a slight reduction of protein expression (Figure S4, right), suggesting a major role of this gene in cell proliferation. Quite similar results were obtained after PLXNB2 silencing, whereas, SPRED1 silencing was able to significantly reduce cell proliferation only in GSCs derived from patient #275 at both surgeries. To analyze the effects of silencing on clonogenic ability, shLAT1, shPLXNB2, shSPRED1, and shNTC-transduced GSCs were plated and grown as single cells. LAT1 silencing significantly reduced the clonogenic ability in GSC#83 and #275Bis compared to the shNTC GSCs (Figures 4A–4D, middle). Furthermore, a significant decrease in the migration of the silenced cells compared to control was found for all the three targets only in GSC#83, whereas for the other three GSC lines we observed a significant decrease of migration ability only in shLAT1 GSC#1, shPLXNB2 GSC#275, and shSPRED1 GSC#275Bis (Figures 4A–4D, right), confirming the different contribution of miR-126 targets in tumorigenic features of GSCs.

A40s-miR-126-3p chimera targets LAT1, PLXNB2, and SPRED1 and inhibits GSC growth

It is widely recognized that aptamers are highly selective carriers for therapeutic agents, including ncRNAs, targeting specific cells.[31–33] Due to the efficient internalization of A40s by target cells, we generated a molecular chimera, A40s-miR-126-3p, by fusing the miR-126-3p to the A40s aptamer (Figures 5A and 5B). We confirmed the successful annealing of the A40s-miR-126-3p chimera through non-denaturing gel electrophoresis analysis (Figure S5A) and demonstrated its ability to deliver miR-126-3p to GSC#1, expressing the EphA2, and not to U87MG cell line that does not express the A40s target receptor (Figure S5B).

The significant increase of miR-126-3p-relative expression, assessed by real-time PCR after incubation of GSC#1 with A40s-miR126-3p chimera and compared with an unrelated sequence-based chimera as a control, confirmed the binding specificity of A40s to its target receptor (Figure S5C). Interestingly, also treatment of GSC#1 with A40s aptamer alone led to an indirect increased expression of miR-126-3p (Figure S5D), leading to a slighter and not significant reduction in cell proliferation (Figure 5C, left) compared to the stronger decreasing experienced by the chimera; suggesting a synergistic effect of the A40s-miR-126-3p conjugate, as it simultaneosly targets two critical pathways involved in GSC maintenance: the miR-126-3p/LAT1/PLXNB2/SPRED1 axis and the EphA2 signaling pathway. A40s-miR-126-3p *in vitro* treatment of GSC#1 was able to significantly inhibit cell growth compared to both untreated and A40s-treated cells (Figure 5C, left). Although A40s-miR-126-3p treatment was able to inhibit both migration and colony formation abilities of GSC#1, the reduction was not significant (Figure 5C, middle and right images).

Moreover, automated western blot system (WES) analysis performed on GSC#1 treated with A40s-miR-126-3p conjugate, revealed a decreased expression of all the three miR-126-3p targets compared to both untreated (CNTR) and A40s-treated cells (Figure 5D), though at different levels (10%, 50%, and 40% reduction of LAT1, PLXNB2, and SPRED1 expression, respectively, in A40s-miR-126-3p chimera-treated cells compared to A40s-treated cells).

Altogether these results confirmed that restoration of miR-126, achieved with A40s-miR-126-3p, reproduces results similar to miR-126 overexpression obtained by lentiviral infection, leading to the chance to use systemic administration of A40s aptamer to specifically deliver miR-126-3p into stem-like cell compartment of GBM.

Aptamer-based miR-126-3p restoration significantly decreases *in vivo* tumor growth

To confirm our findings *in vivo*, the GFP^+ GSC#1 line was grafted into the striatum of severe combined immunodeficiency (SCID) mice (Figure 6A). One week after grafting, mice were randomly assigned to three groups: saline-treated (CNTR), A40s-aptamer alone (A40s), or A40s-miR-126-3p chimera (A40s-miR-126-3p)-treated groups. Intravenous (i.v.) treatment was performed three times a week for three weeks and mice were sacrificed four additional weeks after the end of treatment. A40s-miR-126-3p-treated tumors were smaller and less infiltrating than those treated either with saline or with A40s (Figures 6A, S6A, and S6B). H&E staining and immunostaining for GFP of sagittal mouse brain sections showed a significant reduction of tumor volume in mice treated with A40s-miR-126-3p compared with both mice treated with saline and A40s (both

Figure 4. miR-126 target silencing impairs tumorigenic properties of GSCs

Functional *in vitro* assays on (A) GSC#1 (B) GSC#83, (C) GSC#275, and (D) GSC#275Bis transduced with shNTC, shLAT1, shPLXNB2, or shSPRED1 vectors. Growth curves of GSCs transduced with shNTC or shLAT1, shPLXNB2, shSPRED1 vectors (left). Points and range lines at each day represent mean ± SD of at least two independent experiments in triplicate. Colony formation assay of GSCs transduced with shNTC or shLAT1, shPLXNB2 and shSPRED1 vectors (middle). Percent colony number values from two independent experiments in triplicate were calculated over the correspondent control vector and are shown as mean ± SD for each GSC line. Migration assay of GSCs transduced with shNTC or shLAT1, shPLXNB2 and shSPRED1 vectors (right). Values are reported as percentage relative to control vector and shown as mean ± SD from two independent experiments in triplicate. ns, not significant; *$p < 0.05$' **$p < 0.01$; ***$p < 0.001$ based on Student's *t* test.

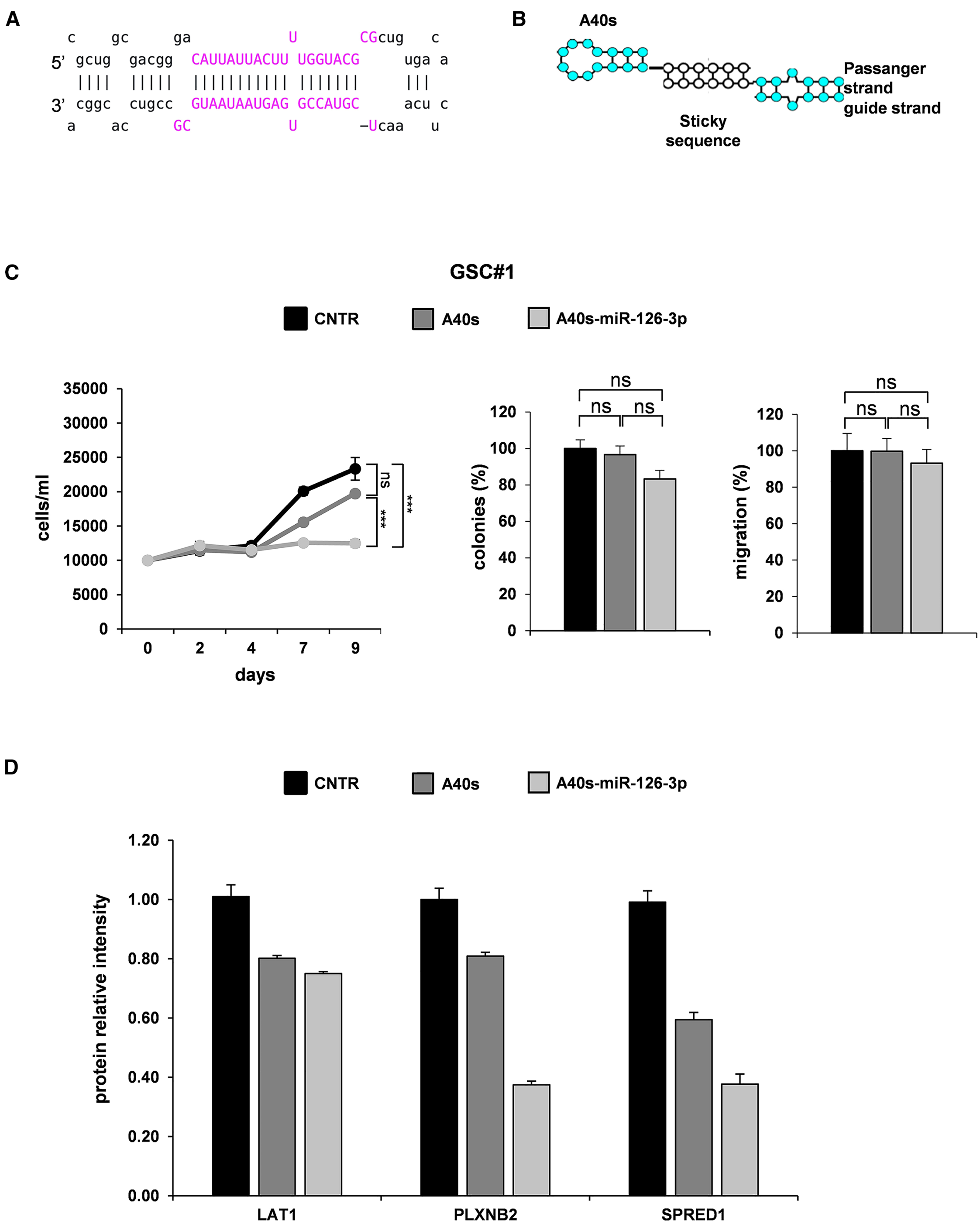

Figure 5. Aptamer A40s-miR-126 impairs tumorigenic abilities of GSCs

(A) Secondary structure of human pre-miR-126 predicted by miRBase. The mature miRNA sequence is highlighted in pink. (B) Scheme of the A40s-miR-126-3p chimera with icons adapted from Bioicons (credit to DBCLS). (C) Functional *in vitro* assays: growth curve (left), colony formation assay (middle), and migration assay (right) of no treated GSC#1 (CNTR), GSC#1 incubated with A40s-aptamer alone (A40s) or A40s-miR-126-3p chimera (A40s-miR-126-3p). Values shown are mean ± SD from two independent experiments in triplicate. ns, not significant; ***$p < 0.001$ based on Student's *t* test. (D) WES analysis of LAT1, PLXNB2, and SPRED1 in no treated GSC#1 (CNTR), GSC#1 incubated with A40s-aptamer alone (A40s) or A40s-miR-126-3p chimera (A40s-miR-126-3p). The protein expression was quantified using AUC (area under the curve) measurements generated using Compass Software. Signal intensity was normalized to the β-actin expression in the same samples.

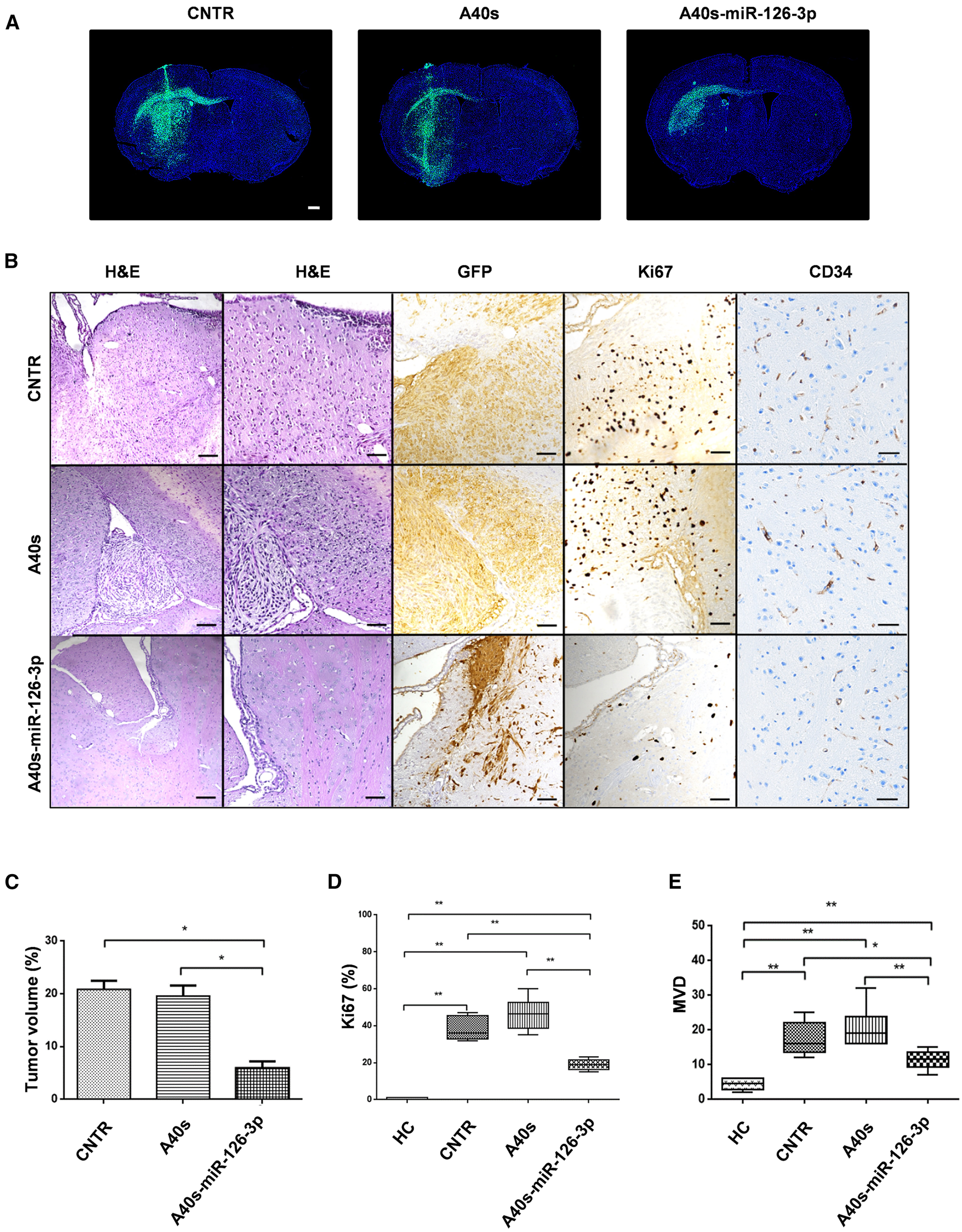

Figure 6. Systemic administration of the A40s-miR-126-3p chimera inhibits orthotopic glioblastoma growth *in vivo*

(A) Representative coronal brain sections collected 8 weeks after intracranial implantation of GFP-positive GSC#1 cells. One week after tumor implantation, mice were randomly assigned to three treatment groups receiving saline (CNTR), A40s aptamer alone (A40s), or the A40s-miR-126-3p chimera (A40s-miR-126-3p). Brain sections were counterstained with DAPI (blue). Scale bars, 500 μm. (B) Representative sagittal brain sections showing H&E staining (100× and 200× magnification), GFP immunostaining to identify tumor cells (200×), Ki67 immunostaining to assess tumor cell proliferation (200×), and CD34 immunostaining to evaluate micro=vessel density (400×). Scale bars: 200 μm, 100 μm, and 50 μm, as indicated. (C) Quantitative morphometric analysis data expressed as the percentage of the tumor volume relative to the total volume of the mouse brain, determined on serial histological brain sections, demonstrating a significant reduction in tumor size in mice treated with the A40s-miR-126-3p chimera

(legend continued on next page)

p = 0.026; Mann-Whitney U test), (Figures 6B and 6C). A40s-miR-126-3p-treated tumors displayed also a significant reduction of proliferation index as assessed by immunostaining for Ki67 compared to both saline and A40s-treated tumors (both p = 0.0022; Mann-Whitney U test), (Figure 6D). To verify whether A40s-miR-126-3p treatment was able to interfere with tumor vascularization, we analyzed in the same sagittal sections, the micro-vessel density (MVD) by immunostaining for human CD34. Treatment with A40s-miR126-3p significantly decreases MVD in tumors compared with those treated both with saline and with A40s (p = 0.026 and p = 0.0022, respectively) reducing it to levels not significantly different from those observed in normal mice (Figure 6E).

To verify the efficacy of miR-126-3p aptamer-mediated delivery, we analyzed miR-126-3p expression by real-time PCR analysis of miR-126-3p levels in mice brain grafted with GFP^+ GSC#1. Figure 7A shows a significant increase of miR-126-3p expression in mouse brains treated with A40-miR-126-3p compared to those treated with saline and A40s.

In addition, the specific delivery of miR-126-3p into tumor mass was further confirmed by *in situ* hybridization in sagittal mouse brain sections, 8 weeks after intracranial grafting, four weeks after the end of treatment, using Purkinje cells of a healthy mouse brain section as positive control (Figure 7B, top images). *In situ* hybridization showed the higher expression of miR-126-3p in sections derived from A40s-miR-126-3p chimera-treated mice compared to those derived from both saline and A40s-treated mice. The successful delivery of miR-126-3p was further confirmed by the decrease of PLXNB2 target expression in sections obtained from A40s-miR-126-3p chimera-treated mice (Figure 7B, bottom images). Quantitative analysis of the *in situ* hybridization and immunohistochemical staining confirmed the histological observations. Tumors treated with the A40s-miR-126-3p chimera showed a significant increase in the percentage of miR-126-positive tumor cells, accompanied by a significant reduction in the percentage of PLXNB2-positive cells compared to the control groups (Figure 7C). The expression of the other two miR-126-3p targets, LAT1 and SPRED1, did not significantly change (data not shown).

Altogether, these *in vivo* results demonstrate the ability of A40s-conjugate to efficiently and selectively deliver miR-126-3p into GBM cells, reaching GSC component located even within invasive niches and protected by the BBB.

DISCUSSION

GBM remains one of the most therapy refractory solid malignancies due to its profound i.t. heterogeneity, highly infiltrative behavior, and the persistence of GSCs, which sustain tumor propagation, therapeutic resistance, and recurrence. In this context, the identification of molecular vulnerabilities capable of simultaneously targeting stemness, invasion, and tumor adaptability represents a major unmet need in neuro-oncology. In the present study, we demonstrate that: (1) miR-126 expression inversely correlates with GBM patients survival; (2) restoration of miR-126 expression exerts tumor-suppressive activity in patient-derived GSC models; and (3) targeted delivery of miR-126 through an A40s aptamer-conjugate effectively suppresses tumor growth and infiltration *in vivo*. Collectively, our findings support a dual conceptual advance: first, the identification of miR-126 as a critical regulator of GSC aggressiveness; and second, the feasibility of exploiting aptamer-mediated delivery systems to selectively target GBM stem-like cell populations.

Our results reinforce previous observations suggesting that miR-126 acts predominantly as a tumor suppressor in GBM biology. Earlier studies reported reduced miR-126 expression in GBM tissues compared with non-neoplastic brain tissue and suggested an association between preserved i.t. miR-126 expression and improved patient outcome.[13] In agreement with these observations, consistent associations were observed in two partially overlapping but separately analyzed cohorts: patients with low miR-126-3p expression in GBM tissues and patients whose derived GSC cultures displayed low miR-126-3p expression both experienced shorter overall survival. Importantly, the prognostic significance observed in our cohort strengthens the hypothesis that miR-126 loss is not merely an epiphenomenon of tumor progression but may actively contribute to the acquisition of aggressive biological traits.

Functional restoration of miR-126 in multiple patient-derived GSC lines induced a marked reduction in proliferative capacity, clonogenicity, and migratory behavior, although with variable magnitude across different cell lines. In particular, GSC#275, established from a treatment-naive primary GBM, exhibited a more limited response to miR-126-3p restoration than GSC#275Bis, which was derived from the recurrent tumor of the same patient after standard chemoradion. Although the molecular mechanisms underlying this difference were not investigated, GBM recurrence is known to be accompanied by extensive genetic, epigenetic and transcriptional evolution driven by therapeutic selective pressure. It is therefore conceivable that treatment-induced molecular remodeling may alter the dependency of recurrent tumors on miR-126-3p-regulated signaling pathways. While this hypothesis requires dedicated investigation, our findings further highlight the remarkable biological heterogeneity of GBM and reinforce the importance of evaluating novel therapeutic strategies across multiple independent patient-derived models, including matched primary and recurrent tumors.

compared with both the saline-treated (CNTR) and A40s-treated groups (both p = 0.026; Mann-Whitney U test). (D) Quantitative analysis of the Ki67 labeling index, expressed as the percentage of Ki67-positive tumor cells, showing a significant reduction in tumor cell proliferation following treatment with the A40s-miR-126-3p chimera compared with both control groups (both p = 0.0022; Mann-Whitney U test). (E) Quantitative analysis of micro-vessel density (MVD), determined by CD34 immunostaining, showing a significant reduction in tumor vascularization in mice treated with the A40s-miR-126-3p chimera compared with both CNTR and A40s-treated mice (p = 0.026 and p = 0.0022, respectively; Mann-Whitney U test).

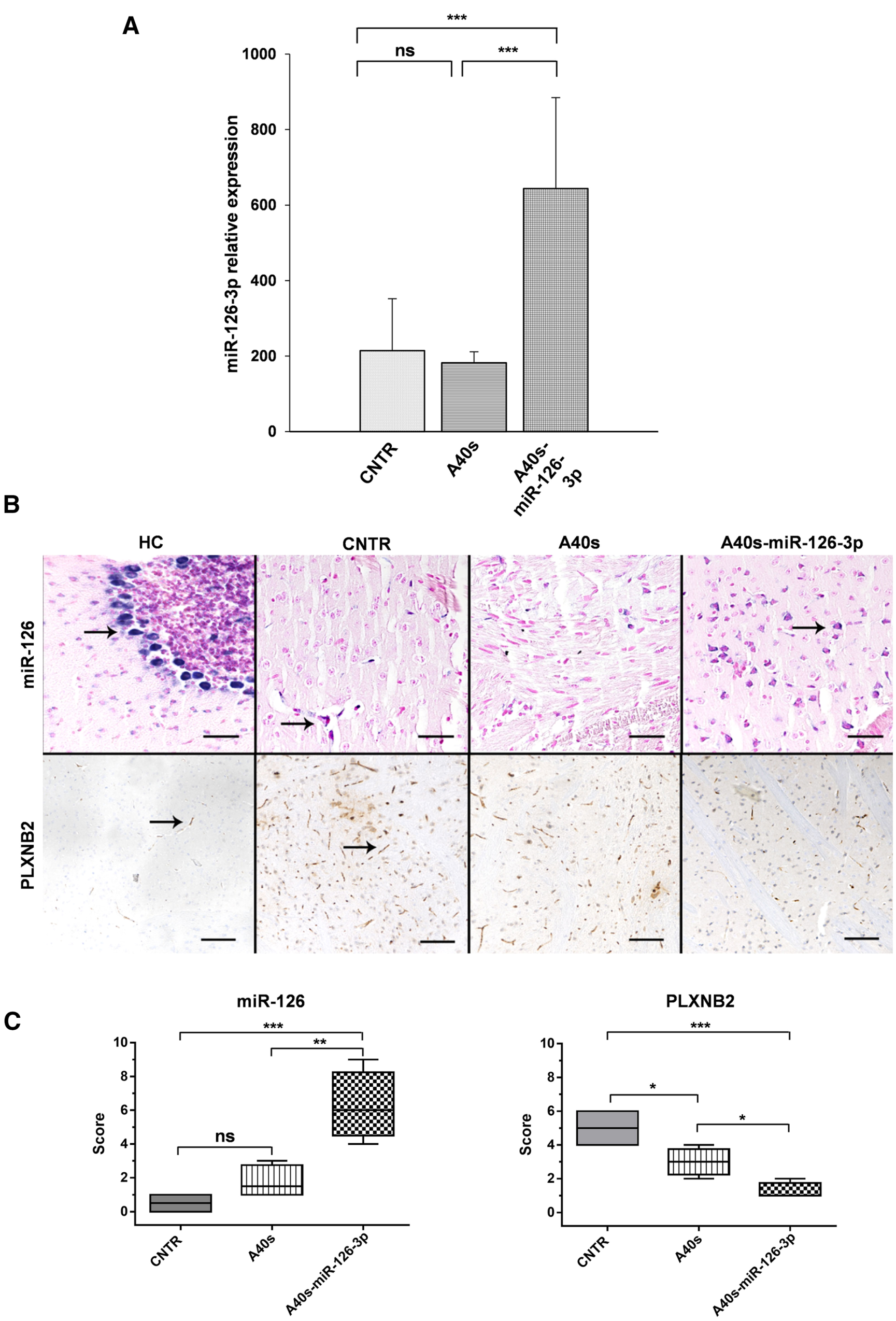

Figure 7. Aptamer-miR-126 treatment restores miR-126-3p expression in GSC-derived brain tumor
(A) RT-PCR analysis of miR-126-3p levels in mice grafted with GSC#1 and treated with saline (CNTR), A40s, and A40s-miR-126-3p. Statistical analysis was performed using two-way ANOVA representing duplicates measurements from the three analyzed groups. Bars report mean ± SD (ns, not significant, ***$p \leq 0.001$). (B) Representative images of sagittal mouse brain sections at 8 weeks after the intracranial injection of GFP^{+} GSC#1. The top images show the expression of miR-126-3p using *in situ* hybridization (nuclear and cytoplasmic blue staining, 200× magnification; Scale bars, 100 μm) in sagittal section of xenograft mouse brain of healthy control (HC, Purkinje cells), mice grafted with GSC#1, treated with saline (CNTR), A40s, and A40s-miR126-3p, respectively. Arrows indicate the representative positive cells. The bottom images show the expression of PLXNB2 using immunohistochemistry (cytoplasmatic brown staining, 100× magnification; Scale bars, 200 μm) in sagittal section of xenograft mouse brain of healthy control (HC, normal brain), mice grafted with GSC#1 and treated with saline (CNTR), A40s and A40s-miR-126-3p, respectively. Arrows indicate positive cells. (C) Quantitative analyses of miR-126 expression (left) and PLXNB2 expression (right) are reported as a combined score based on the percentage of positive cells and staining intensity. Statistical analysis was performed using one-way ANOVA with multiple comparison by Tukey's test, ns, not significant; *$p < 0.05$, **$p < 0.01$, ***$p < 0.001$.

Mechanistically, our data identify LAT1, PLXNB2, and SPRED1 as relevant downstream mediators of miR-126 activity in GSCs. An interesting observation emerging from our study is the partial discordance between the modulations of target mRNA and the corresponding protein levels following miR-126-3p restoration. Although LAT1, PLXNB2, and SPRED1 transcripts exhibited heterogeneous or relatively modest changes across the different GSC models, protein expression was consistently reduced. This observation is in agreement with the well-established mechanism of action of miRNAs, which regulate gene expression through both mRNA destabilization and translational repression. The relative contribution of these mechanisms is highly target- and context-dependent and may explain why protein modulation was more evident than transcript changes in our experimental models. Furthermore, additional post-transcriptional and post-translational regulatory mechanisms cannot be excluded and may also contribute to the observed differences between mRNA and protein expression. The simultaneous modulation of these targets suggests that miR-126 does not act through a single linear pathway but rather through coordinated repression of multiple biological programs involved in metabolic adaptation, invasive capacity, and signaling plasticity.

Among these targets, LAT1 appears particularly relevant in the context of GBM metabolic reprogramming.[34] LAT1 is a high-affinity transporter for large neutral amino acids and is critically involved in sustaining anabolic metabolism and mTOR activation in highly proliferative cancer cells. Increased LAT1 expression has been associated with aggressive glioma phenotypes, enhanced proliferation, metabolic flexibility, and poor prognosis. By repressing LAT1 expression, miR-126 may interfere with the metabolic demands required for

maintenance of the stem-like compartment, thereby contributing to the reduction in proliferation and clonogenic potential observed in our models.

Equally relevant is the identification of PLXNB2 as a miR-126-regulated target. PLXNB2 has recently emerged as a critical mediator of glioma cell invasion and adaptation to confined migratory environments.[16] Recent studies demonstrated that PLXNB2 regulates membrane tension dynamics, endocytosis, cytoskeletal polarization, and mechano-electrical coupling required for GBM cells to migrate through spatially restricted environments within the brain parenchyma.[28] The highly infiltrative nature of GBM represents one of the principal causes of therapeutic failure and recurrence; therefore, inhibition of molecules involved in invasive adaptation has substantial therapeutic implications. Our data showing that miR-126 downregulates PLXNB2 expression and significantly impairs GSC migratory capacity strongly suggest that suppression of invasive programs may represent a central component of miR-126 antitumoral activity. Notably, the reduction in migration observed following PLXNB2 silencing supports the biological relevance of this axis and aligns with emerging evidence implicating Plexin-B2 in GBM dissemination and poor clinical outcome.

The role of SPRED1 in our model appears more complex and potentially context-dependent. SPRED1 is traditionally regarded as a negative regulator of the RAS/mitogen-activated protein kinase (MAPK) signaling cascade[35]; however, increasing evidence suggests that its biological function may vary according to cellular context and oncogenic background.[36] In our experimental setting, modulation of SPRED1 contributed to alterations in GSC behavior, suggesting that miR-126-mediated regulation of signaling homeostasis may involve intricate compensatory mechanisms.

Importantly, silencing experiments demonstrated that inhibition of LAT1, PLXNB2, and SPRED1 partially reproduced the phenotypic effects induced by miR-126 restoration, although no single target completely recapitulated the full spectrum of biological alterations observed after miR-126 overexpression. The observation that individual small hairpin RNA (shRNA)-mediated silencing produced a stronger antiproliferative effect than miR-126 restoration may initially appear counterintuitive. However, this finding is consistent with the distinct mechanisms of action of miRNAs and gene-specific RNA interference. Whereas shRNAs are designed to induce robust and sustained suppression of a single target gene; miRNAs generally fine-tune the expression of multiple transcripts, resulting in partial repression of each individual target while coordinately modulating entire signaling networks. Therefore, the biological activity of miR-126-3p is likely to derive from the combined regulation of multiple downstream effectors rather than complete inhibition of a single oncogenic pathway.

Efficient and targeted-delivery remains one of the principal limitations of RNA-based therapeutics in neuro-oncology, particularly because of BBB constraints, systemic instability, and off-target toxicity. Aptamers have emerged as highly promising delivery vehicles due to their low immunogenicity, high target specificity, favorable tissue penetration, and potential capability to cross the BBB.[18] Previous studies identified the A40s aptamer as a selective ligand for GSCs through recognition of EphA2-positive stem-like populations and demonstrated its ability to internalize therapeutic cargo into GBM cells. Building upon these observations, our study demonstrates that conjugation of miR-126 to the A40s aptamer allows selective intracellular delivery of the therapeutic miRNA while preserving biological activity.[19,20]

Importantly, treatment with the A40s-miR-126 chimera resulted in increased intracellular miR-126 levels together with downregulation of relevant downstream targets, confirming the functional integrity of the conjugate after cellular internalization. *In vitro*, the aptamer-miRNA construct significantly reduced proliferation and clonogenic growth of GSCs, indicating that selective delivery of miR-126 is sufficient to induce biologically relevant antitumoral effects.

The *in vivo* findings further strengthen the translational significance of this approach. Systemic administration of the A40s-miR-126 conjugate in orthotopic xenograft models resulted in reduced tumor burden, lower proliferative activity, and decreased microvascular density. Particularly noteworthy is the reduction in infiltrative behavior observed in treated tumors. Since diffuse invasion into surrounding brain tissue is a hallmark of GBM and a major obstacle preventing complete surgical eradication, therapeutic strategies capable of limiting invasion may have profound clinical implications. The anti-invasive effect observed *in vivo* is consistent with the suppression of PLXNB2-mediated migratory programs and suggests that miR-126 restoration may simultaneously target both tumor expansion and infiltrative dissemination.

The observed reduction in microvascular density additionally supports the antiangiogenic activity of miR-126 previously described in GBM models. Prior studies demonstrated that miR-126 negatively regulates VEGF-related pathways and modulates endothelial interactions within the tumor microenvironment.[37] Given the central role of aberrant angiogenesis in GBM progression, the ability of miR-126 to impair vascular support may further contribute to its therapeutic efficacy. Interestingly, antiangiogenic therapies such as bevacizumab often induce adaptive invasive phenotypes in GBM.[38] In this regard, the simultaneous antiangiogenic and anti-invasive effects mediated by miR-126 restoration may represent a potentially advantageous therapeutic combination capable of limiting adaptive escape mechanisms.

Another important aspect emerging from our study concerns the demonstration that A40s-mediated delivery of miR-126-3p efficiently modulates the malignant phenotype of patient-derived GSCs, supporting the potential of this approach as a targeted therapeutic strategy for GBM. Increasing evidence indicates that GSCs are highly resistant to conventional therapies and are major drivers of recurrence following surgery, radiotherapy, and temozolomide

(TMZ) treatment. Therefore, therapeutic approaches capable of selectively eliminating or functionally suppressing GSCs are considered essential for improving long-term disease control. The use of the A40s aptamer, previously demonstrated to selectively recognize GSC population,[19] provides a biologically rational strategy to preferentially deliver therapeutic cargo to the most aggressive and therapy-resistant cellular compartment within GBM.

Despite the encouraging results obtained in the present study, several limitations should be acknowledged. First, our work represents a preclinical proof-of-concept study evaluating a single aptamer-mediated delivery strategy for the restoration of a single therapeutic miRNA. Whether similar efficacy can be achieved using alternative aptamer platforms or different tumor-suppressive miRNAs remains to be determined. Second, although the antitumor activity of the A40s-miR-126-3p chimera was demonstrated in an orthotopic patient-derived GBM xenograft model, validation in additional preclinical models, including genetically engineered and immunocompetent GBM models, will be necessary to better define its therapeutic potential. Finally, while systemic administration of the chimera produced significant antitumor activity without evidence of overt toxicity during the study period, dedicated pharmacokinetic, biodistribution, and long-term safety studies will be required before clinical translation. Addressing these aspects will be essential for the further development of aptamer-guided miRNA therapeutics for GBM. Nevertheless, the present study provides, to our knowledge, the first demonstration that systemic aptamer-guided delivery of miR-126-3p can efficiently reach orthotopic glioblastoma, restore i.t. miR-126 expression, modulates downstream target proteins, and produce significant antitumor activity *in vivo*. These findings provide a strong rationale for the further preclinical development of this therapeutic platform.

Conclusions

In conclusion, this study provides preclinical evidence that restoration of miR-126-3p represents a promising therapeutic strategy for GBM by simultaneously targeting multiple pathways involved in tumor growth, invasion, and stem-like cell maintenance. Using patient-derived GSC models, we demonstrated that miR-126-3p restoration inhibits malignant phenotypes and modulates key downstream effectors, including LAT1, PLXNB2, and SPRED1. Importantly, conjugation of miR-126-3p to the EphA2-targeting A40s aptamer enabled efficient systemic delivery to orthotopic GBM, resulting in i.t. miR-126-3p restoration, target modulation, and significant antitumor activity *in vivo*.

Overall, our findings establish a strong proof-of-concept for aptamer-guided delivery of tumor-suppressive miRNAs as a precision therapeutic strategy for GBM. Furthermore, beyond the therapeutic potential of miR-126-3p itself, the A40s-based delivery platform described here may provide a versatile framework for the selective systemic delivery of other tumor-suppressive miRNAs or therapeutic RNA molecules to EphA2-positive GBM cells, thereby opening new opportunities for precision RNA therapeutics in neuro-oncology.

MATERIALS AND METHODS

Cell cultures

GSC lines were isolated from surgical samples of adult patients who had undergone craniotomy at the Institute of Neurosurgery, Catholic University of Rome, upon patient informed consent and approval by the local ethical committee (protocol ID: 2253 and 3782). Clinical features of GBM patients and tumors are shown in Table S1. Surgical GBM specimens were subjected to mechanical dissociation and the resulting cell suspension was cultured in a serum free medium supplemented with basic fibroblast growth factor (b-FGF) and epidermal growth factor (EGF) (PeproTech, London, UK) as previously described.[39] GSC lines were validated by short tandem repeat (STR) DNA fingerprinting. Nine highly polymorphic STR loci plus amelogenin (Cell ID System, Promega Inc., Madison, WI, USA) were used. Detection of amplified fragments was obtained by ABI PRISM 3100 Genetic Analyzer (Applied Biosystems, Carlsbad, CA, USA). Data analysis was performed by GeneMapper software, v.4.0 (Biological Bank and Cell Factory, National Institute for Cancer Research, IST, Genoa, Italy).[40] All GSC line profiles were challenged against public databases to confirm authenticity. Clinical features of GSC-generating GBM patients and tumors are previously reported.[41]

The packaging cell line, 293T and the glioma cell line U87MG, were maintained in DMEM (Euroclone, Milan, Italy) supplemented with 10% (v/v) heat-inactivated fetal bovine serum (FBS, Euroclone), 2 mM L-glutamine, 100 U/mL of penicilline and 100 μg/mL of streptomycin (Euroclone). Both cell lines were purchased from ATCC. All cell lines were regularly checked to exclude mycoplasma contamination by Mycoalert Detection Kit (Lonza Walkersville Inc., Walkerswille, MD, USA).

Plasmid constructs and lentivirus infection

The miR-126, a 604-base-pair fragment spanning the miR-126&126*locus precursor, was PCR-amplified from human genomic DNA (NT_024000.16 from nt 347834 to nt 348437) by using AccuPrime Taq DNA polymerase high fidelity (Invitrogen, Carlsbad, CA, USA) and cloned in the 3′ untranslated region (3′-UTR) of RFP in the pTRIPZ doxycycline inducible lentiviral vector (Thermo Fisher Scientific, Waltham, MA, USA) as previously described.[23]

For LAT1, PLXNB2, and SPRED1 silencing, short hairpin shLAT1-GFP (TL309279), shPLXNB2-GFP (TL317033), and shSPRED1-GFP (TL301404) lentiviral vectors were purchased from OriGene Technologies (Rockville, MD, USA).

Lentiviral particles were produced in 293T at 70%–80% confluency, using the calcium phosphate transfection protocol and infection was performed as previously reported.[42]

After infection, for TRIPZ and TRIPZ-miR-126 vectors, transduced cells were selected with puromycin and RFP fluorescence was evaluated by CytoFLEX LX flow cytometer (Beckman Coulter, Milan,

Italy) upon doxycycline induction (Sigma-Aldrich Inc., Saint Louis, MO, USA). For shLAT1, shPLXNB2, and shSPRED1 vectors, GFP fluorescence of transduced cells was evaluated by CytoFLEX LX flow cytometer and RFP- and GFP-positive cells were flow sorted by MoFlo Astrios EQ cell sorter (Beckman Coulter).

RNA extraction and real-time PCR

Total RNA was isolated from cells using TRIzol reagent (Invitrogen by Thermo Fisher Scientific). Reverse transcription of total miRNA was performed starting from equal amounts of total RNA/sample (500 ng) using miScript II reverse transcription kit with HiSpec buffer (QIAGEN, Milan, Italy). Quantitative analysis of miR-126-3p and U6 (as internal reference) was performed by real-time PCR using specific primers (QIAGEN) and miScript SYBR Green PCR Kit (QIAGEN). To detect the expression levels of miR-126-3p in cells treated with A40s-miR-126-3p, $1,5 \times 10^4$ cells were treated with 0.4 μM of A40s-miR-126-3p chimera or control for 72 h.

For *in vivo* analysis, following total RNA extraction the relative amount of miR-126-3p was determined by performing TaqMan Small RNA Assays (Thermo Fisher Scientific) optimized for tumor tissue samples, following the manufacturer's recommendations. U6 small nuclear RNA was used as the endogenous reference control.

Real-time PCR for LAT1, PLXNB2 and SPRED1 mRNA detection was performed with SYBR Green Master Mix (Applied Biosystems by Thermo Fisher Scientific) and normalized with GAPDH. The specific primers were listed in the following table. All reactions were run in duplicate in QuantStudio 12K Flex (Applied Biosystems by Thermo Fisher Scientific). The following primers were used: GAPDH: ACCTGACCTGCCGTCTAG (forward), CCTGCTTCA CCACCTTCT (reverse); LAT1: CGTCCAGATCGGGAAGGGT (forward), CAGCACAATGTTCCCCACATC (reverse); PLXNB2: AGCCTCTTCAAGGGCATCTG (forward), GCCACGAAAGACT TCTCCCC (reverse); SPRED: GCTAACCATCGCAAGCAGAAC (forward), CATTGTATGTCAGACTCAGAGGGAG (reverse).

Cell growth, migration colony formation assays

For the proliferation assay, GSC lines were plated at a density of 2×10^4/mL in 96-well plates in triplicate. For treated GSCs, 0.8 μM of A40s or A40s-miR126-3p chimera were added at day 0 and 0.4 μM of A40s or A40s-miR126-3p chimera were added every 72 h until the end of the assay. Cell proliferation was monitored by using the CellTiter-Blue Viability Assay (Promega).

The motility of GSC lines was evaluated by plating in Corning FluoroBlok Multiwell Inserts System (Corning Life Sciences, Tewksbury, MA, USA), according to manufacturer's instruction. For treated GSCs, 72 h before starting the assay a pre-treatment was performed by adding 0.8 μM of A40s or A40s-miR-126-3p chimera, followed by treatment with 0.4 μM of A40s or A40s-miR126-3p chimera at the beginning of the assay.

Colony formation ability was evaluated by plating a single cell/well in 96-well plates. After 3 weeks, each well was examined and the number of spheres/cell aggregates was counted. For treated GSCs, 0.8 μM of A40s, or A40s-miR-126-3p chimera were added at the beginning of the assay and 0.4 μM of A40s or A40s-miR-126-3p chimera were added every 72 h until the end of the assay.

For TRIPZ and TRIPZ-miR-126 transduced GSCs, all the experiments were performed in stem cell medium in the presence of doxycycline.

miRNA target prediction

Candidate miR-126-3p target genes were identified using TargetScan 7.0 (https://www.targetscan.org), and miRDB (https://www.mirdb.org), which provide computationally predicted and evolutionarily conserved miRNA-mRNA interactions.

Automated capillary WES

Total protein lysates were extracted by using Nonidet-P40 lysate buffer (1% NP40, 200 nM NaCl, 50 mM Tris pH 7.4) with addition of protease and phosphatase inhibitors, incubated for 30 min at 4°C, vortexed and centrifuged at 12,000 rpm for 10 min at 4°C. Protein concentration was measured by BioRad protein-assay (Bio-Rad Laboratories Inc., Hercules, CA, USA). Protein expression analysis was performed using capillary WES technology (ProteinSimple by Bio-Techne, Minneapolis, MN, USA) according to the manufacturer's instruction. 500 ng of sample was mixed with fluorescent 5× Master Mix, maintained for 5 min at 95°C and loaded into a WES 12–230 kDa prefilled plate, along with a biotinylated protein ladder, blocking buffer, primary antibody, ProteinSimple HRP-conjugated anti-rabbit, or anti-mouse secondary antibody, luminol-peroxide, and washing buffer. Immunodetection was performed automatically. Results were reported as virtual gels based on chemiluminescence signals. The acquisition and quantitative analysis of images were performed using AUC (area under the curve) measurements generated by Compass software (ProteinSimple by Bio-Techne). The following primary antibodies were used: LAT1 (TA381705, 1:10, OriGene); PLXNB2 (sc-373969, 1:20), and SPRED1 (sc-393198, 1:10) from Santa Cruz Biotechnology Inc. (Dallas, TX, USA); β-actin (A5441, 1:1000, Sigma-Aldrich Inc).

To detect the levels of miR-126-3p target proteins in cells treated with A40s-miR126-3p, cells were treated with 0.4 μM of A40s or A40s-miR-126-3p chimera every 72 h for 1 week.

Chimera preparation

miRBase (https://www.miRbase.org) was used to predict the secondary structure of human pre-miR-126. 2′-fluoropyrimidine RNAs synthesized by Bio-Fab research s.r.l. (Rome, Italy) were used for chimera production according to previously described protocol.[19] The sequences used for the chimera conjugates were as follows: Guide-miR-126-3p 5′-CUCGUACCGUGAGUAAUAAUGCG-3′; passenger-miR126-3p 5′-GACAUUAUUACUUUUGGUACGCG GGCUAUCUAGAAUGUAC-3′. The negative control was made

by replacing sticky A40s with an unrelated sticky aptamer, Scra stick: 5′UUCGUACCGGGUAGGUUGGCUUGCACAUAGAACGUGU CAXXXXGUACAUUCUAGAUAGCC-3′. All RNA sequences contain 2′F-Py. Stick sequences (underlined) consist of 2′F-Py and 2′-oxygen-methyl purines, X indicates C3 spacer. In detail, to prepare chimeras 10 μM passenger RNA strand and 10 μM guide strand, in the appropriate 10× binding buffer (200 mM HEPES [pH 7.4], 1.5 M NaCl, 20 mM CaCl2), were first denatured at 95°C for 15 min, subsequently brought to 55°C for 10 min, and finally warmed up to 37°C for 20 min. The annealed passenger and guide strand thereby obtained was combined with sticky A40s or control aptamer (ratio 1:1) and kept 30 min at 37°C to allow for the annealing of the stick sequences. The correct annealing was assessed by 12% non-denaturing polyacrylamide gel in TAE (Tris-Acetate-EDTA) followed by gel staining with SYBR safe.

Intracranial implantation of GSCs into immunocompromised mice followed by aptamer-based treatment

Animal experiments were performed in accordance to relevant institutional and national regulations (Aut.n. 53/2023-PR, prot. D9997.150). Thirty non-obese diabetic (NOD)-SCID mice (male; 4–6 week old; Charles River, Italy) were implanted intracranially with 2×10^5 GFP-expressing GSC#1 resuspended in 5 μL of serum-free medium. For brain grafting, the mice were anesthetized with intraperitoneal (i.p.) injection of diazepam (2 mg/100 g) followed by intramuscular (i.m.) injection of ketamine (4 mg/100 g). Animal skulls were immobilized in a stereotactic head frame and a burr hole was made 2 mm right of the midline and 1 mm posterior to the coronal suture, and cells were slowly injected using the tip of a 10 μL Hamilton microsyringe placed at a depth of 3 mm from the dura. One week after grafting, the mice were randomly allocated to three experimental groups (n = 10 per group). Four animals per group were used for immunofluorescence studies, four for histopathological, immunohistochemical, and *in situ* hybridization analyses, and two for quantitative reverse-transcription PCR (RT-qPCR) evaluation of tumor tissues and treated according to the protocol described in Table S2.

After 8 weeks from grafting, animals were deeply anesthetized and transcardially perfused with 0.1 M PBS (pH = 7.4), followed by 4% paraformaldehyde in 0.1 M PBS.

Brains were removed, cryoprotected by sequential incubation with 15% and then 30% sucrose solutions, both overnight at 4°C, and serially cryotomed at 25 μm on the coronal plane.

Free-floating brain slices for the different experimental groups were analyzed for the presence and distribution of GFP$^+$ GSCs. All sections were labeled with DAPI for nuclei segmentation (excitation wavelength used for imaging 391 nm); sections were mounted on slides and coverslipped with antifade medium (ProLong Glass mountant, Invitrogen). Images of each brain slice were acquired using a 10× or a 20/0.8 NA objective on a Leica THUNDER Imager DMI8 fluorescence microscope (Leica Microsystems, Wetzlar, Germany). A tile scan function was used to rapidly image the entire brain slice using the Leica LASX software.

The images were subjected to small volume computational clearing (SVCC) using LASX software. The cranio-caudal extension of the brain area invaded by GFP$^+$ GSCs was assessed on serial coronal sections. The volume of the brain invaded by the tumor (V) was determined according to the equation, $V = A\ x\ b$, where A is the mean of the tumor area calculated on coronal sections through the ImageJ software, Fiji and b is the cranio-caudal extension of the tumor. The cranio-caudal extension was estimated by counting the consecutive coronal sections containing GFP-positive tumor cells and multiplying this number by the section thickness (25 μm).

Immunohistochemistry, MVD evaluation and *in situ* hybridization

Briefly, 4-μm-thick sections from FFPE (formalin-fixed paraffin-embedded) blocks were mounted on silanized slides, deparaffinized, and rehydrated before undergoing microwave-based antigen retrieval (3 × 5 min at 750 W) in pH 6.0 citrate acid buffer. After the inhibition of endogenous peroxidase with 3% H_2O_2 for 10 min, the sections were incubated with the following pre-diluted primary antibodies: anti-Ki67 (clone 30–9) rabbit monoclonal (Roche), or anti-GFP (clone 168AT1211) mouse monoclonal (Abcepta, Inc. San Diego CA), or anti-GFAP (clone EP672Y) rabbit monoclonal (Sigma-Aldrich), or anti-CD34 (clone QBEnd 10) mouse monoclonal (Dako), or anti-LAT1 (TA381705) rabbit polyclonal (Origene), or anti-SPRED1 (clone E−5) mouse monoclonal (Sigma-Aldrich), or anti-PLXNB2 (clone A-5) mouse monoclonal antibody (Sigma-Aldrich). Primary antibodies were detected using the UltraTek HRP Anti-polyvalent avidin-biotin-peroxidase kit (ScyTek, USA), according to the manufacturer's instructions. Signal localization was visualized with 3,3′-diaminobenzidine tetrahydrochloride, while Mayer's hematoxylin served as the nuclear counterstain. Appropriate positive control tissues were processed in parallel under identical experimental conditions. Negative controls were obtained by substituting the primary antibody with PBS (pH 7.4) or an isotype-matched non-immune immunoglobulin. Two pathologists, blinded to the clinicopathological data, performed independent evaluations. Any inter-observer variation greater than 5% was re-examined and resolved by consensus using a double-headed microscope. To evaluate immunohistochemical expression, a combined score based on the percentage of positive cells and staining intensity was used. The score for the extent of the immunohistochemistry (IHC)-stained area was scaled as 0 for no IHC signal, 1 for 1%–30%, 2 for 31%–70%, and 3 for 71%–100% of tumor cells stained. The score for IHC intensity was also scaled as 0 for no IHC signal, 1 for weak, 2 for moderate, and 3 for strong IHC signals. The final score (ID-score) used in the analysis was calculated by multiplying the extent and intensity scores, with a maximum score equal to 9; the ID scores 0–3 and 4–9 discriminate samples with low or high expression, respectively. MVD was measured according to the method previously described with few modifications.[41] Briefly, in areas of most intense neovascularization, individual micro-vessel counts (using

CD34 staining) were made on a 200× magnification field (equivalent to 0.7386 mm^2). Any endothelial cell or endothelial cell cluster was considered as a single countable micro-vessel. MVD was expressed as the mean number of micro-vessels per field from 3 highly vascularized areas in each case. Tumor volume in mouse brain xenografts was evaluated as following. Briefly, brains were harvested, fixed in formalin, and serially sectioned at predefined intervals. Tumor areas were delineated on each anti-GFP immunostaining brain section and quantified using digital image analysis software (ImageJ, National Institutes of Health, USA) after appropriate calibration. Total tumor volume was calculated by summing the tumor areas across all sections and multiplying by the section thickness and the sampling interval, according to the Cavalieri principle. Data were expressed as a percentage of the tumor volume relative to the total volume of the mouse brain. All measurements were performed in a blinded fashion by two independent observers to ensure accuracy and reproducibility.

For the detection of miR-126-3p, chromogenic *in situ* hybridization (CISH) was performed using the miRCURY LNA miRNA ISH Optimization Kit (QIAGEN), following the manufacturer's instructions. The assay includes three digoxigenin (DIG)-labeled probes: a double-DIG-labeled (5′ and 3′) probe targeting the mature hsa-miR-126-3p sequence (UCGUACCGUGAGUAAUAAUGCG), a 5′-DIG-labeled U6 small nuclear RNA (snRNA) probe as positive control, and a double-DIG-labeled (5′ and 3′) scramble-miR probe as negative control. Briefly, 6-μm-thick FFPE sections were mounted on silanized slides and then incubated at 60°C for 45 min. Deparaffinized sections underwent miRNA demasking through Proteinase K treatment at 37°C for 10 min in a hybridization chamber, followed by PBS washes. Hybridization was conducted using 40 nM of the miR-126-3p detection probe, properly diluted in hybridization buffer, at 50°C for 1 h, followed by standardized stringency washes. After blocking, DIG-labeled hybrids were detected via an alkaline-phosphatase-conjugated anti-DIG antibody (1:800, 1 h at repetition time, RT) and chromogenically developed with the substrate 4-nitro-blue tetrazolium/5-bromo-4-chloro-3′-indolylphosphate (NBT/BCIP) and levamisole. Slides were then counterstained with nuclear fast red, dehydrated, and mounted with Eukitt medium. Cover-slipped slides were left to dry overnight before microscopic evaluation. Colorectal cancer and healthy brain tissues acted as positive and negative biological controls for miR-126-3p, respectively. For the technical negative controls, PBS was used in place of the primary antibody or probe. Tissue sections were examined under a light microscope. Specifically, the expression of miR-126-3p appeared as blue staining in the nuclei and cytoplasm and was quantified by assessing staining intensity and the percentage of positive cells.

To evaluate PLXNB2 and miR-126 expression, a combined score based on the percentage of positive cells and staining intensity was used. The score for the extent of the stained area was scaled as 0 for no signal, 1 for 1%–30%, 2 for 31%–70%, and 3 for 71%–100% of tumor cells stained. The score for intensity was also scaled as 0 for no signal, 1 for weak, 2 for moderate, and 3 for strong signals. The final score (ID-score) used in the analysis was calculated by multiplying the extent and intensity scores, with a maximum score equal to 9; the ID scores 0–3 and 4–9 discriminate samples with low or high expression, respectively.

Statistical analysis

Statistical analysis was performed using GraphPad-Prism 5 software (GraphPad software, www.graphpad.com) and MedCalc software (MedCalc software, https://www.medcalc.org/en/).

For Kaplan-Meier survival analyses, miR-126-3p expression was evaluated separately in the GBM tissue and patient-derived GSC cohorts. Within each cohort, the mean miR-126-3p expression value was used as the cutoff to stratify samples into high- and low-expression groups. Samples with expression values above the cohort-specific mean were classified as high-expressing, whereas those with values below the mean were classified as low-expressing. The two cohorts partially overlapped, as approximately 30% of the GSC cultures were derived from tumors also included in the tissue cohort. Differences in survival between groups of patients and GSCs were compared using the log-rank test. A statistical comparison of continuous variables between groups was performed using the Student's t test, Mann-Whitney U test, one-way or two-way ANOVA, as appropriate. Categorical variables were compared using statistic chi-squared test and Fisher's exact test. Only values of $p < 0.05$ were considered as significant and the level of significance is indicated in the plots using asterisks as follows: $^{*}p < 0.05$, $^{**}p < 0.01$, and $^{***}p < 0.001$.

DATA AND CODE AVAILABILITY

The datasets used and/or analyzed during the current study are available from the corresponding author upon reasonable request.

ACKNOWLEDGMENTS

This work was supported by Associazione Italiana per la Ricerca sul Cancro, AIRC (IG2021 n.26515) to L.R.-V. This work has been partially conducted under the National Plan for Complementary Investments to the NRRP, project "D34H—Digital Driven Diagnostics, prognostics and therapeutics for sustainable Health care" (project code: PNC0000001), Spoke 3, funded by the Italian Ministry of University and Research and under the PNRRTR1-2023-12377972 project "Biobanking, Biomarkers and Big Data: B3D for personalized treatment of glioblastoma" (M6C2I2.1), funded by Italian Ministry of Health. *In memory of Prof. Roberto Pallini, MD.* The authors dedicate this work to the memory of Roberto Pallini, an outstanding neurosurgeon and scientist who devoted his career with extraordinary dedication to glioblastoma research. Beyond his invaluable scientific contributions, he was for all of us an inspiring mentor, an illustrious master, and a guide whose legacy will continue to shape our work and our vision of translational neuro-oncology.

AUTHOR CONTRIBUTIONS

L.R.-V. conceived and designed the study and wrote the manuscript; M. Buccarelli and G. Castellani took care of GSC cultures and carried out in vitro assays; A.A. took care of chimera preparation; Q.G.D'A., G.D.L., and M.O. performed brain xenografts; M.M., M. Ballato, and G.R. performed ISH/IHC and data analysis of in vivo experiments; C.M. performed confocal and fluorescence microscopy analysis of brain xenograft sections; A.B. carried out cell sorting and flow cytometry analysis; F.P. performed automated capillary western immonoassays; R.I. took care of molecular assays (real-time PCR, plasmid constructs); A.D.G. performed quantitative analysis included in the revised version; N.F., G.M., M. Biffoni, M.C., and G. Condorelli contributed to the supervision and critical revision of the manuscript. All the authors discussed the results, provided critical feedback and contributed to revise the manuscript.

DECLARATION OF INTERESTS

The authors declare no competing interests.

SUPPLEMENTAL INFORMATION

Supplemental information can be found online at https://doi.org/10.1016/j.omtn.2026.103103.

REFERENCES

1. Louis, D.N., Perry, A., Wesseling, P., Brat, D.J., Cree, I.A., Figarella-Branger, D., Hawkins, C., Ng, H.K., Pfister, S.M., Reifenberger, G., et al. (2021). The 2021 WHO Classification of Tumors of the Central Nervous System: a summary. Neuro Oncol. *23*, 1231–1251. https://doi.org/10.1093/neuonc/noab106.

2. Wu, W., Klockow, J.L., Zhang, M., Lafortune, F., Chang, E., Jin, L., Wu, Y., and Daldrup-Link, H.E. (2021). Glioblastoma multiforme (GBM): An overview of current therapies and mechanisms of resistance. Pharmacol. Res. *171*, 105780. https://doi.org/10.1016/j.phrs.2021.105780.

3. Satish, S., Athavale, M., and Kharkar, P.S. (2024). Targeted therapies for Glioblastoma multiforme (GBM): State-of-the-art and future prospects. Drug Dev. Res. *85*, e22261. https://doi.org/10.1002/ddr.22261.

4. Shang, R., Lee, S., Senavirathne, G., and Lai, E.C. (2023). microRNAs in action: biogenesis, function and regulation. Nat. Rev. Genet. *24*, 816–833. https://doi.org/10.1038/s41576-023-00611-y.

5. Balachandran, A.A., Larcher, L.M., Chen, S., and Veedu, R.N. (2020). Therapeutically Significant MicroRNAs in Primary and Metastatic Brain Malignancies. Cancers (Basel) *12*, 2534. https://doi.org/10.3390/cancers12092534.

6. Han, F., Huang, D., Meng, J., Chu, J., Wang, M., and Chen, S. (2022). miR-126-5p enhances radiosensitivity of lung adenocarcinoma cells by inhibiting EZH2 via the KLF2/BIRC axis. J. Cell Mol. Med. *26*, 2529–2542. https://doi.org/10.1111/jcmm.17135.

7. Msheik, Z.S., Nassar, F.J., Chamandi, G., Itani, A.R., Gadaleta, E., Chalala, C., Alwan, N., and Nasr, R.R. (2022). miR-126 Decreases Proliferation and Mammosphere Formation of MCF-7 and Predicts Prognosis of ER+ Breast Cancer. Diagnostics *12*, 745. https://doi.org/10.3390/diagnostics12030745.

8. Zhang, Y., Qin, X., Jiang, J., and Zhao, W. (2020). MicroRNA-126 exerts antitumor functions in ovarian cancer by targeting EGFL7 and affecting epithelial-to-mesenchymal transition and ERK/MAPK signaling pathway. Oncol. Lett. *20*, 1327–1335. https://doi.org/10.3892/ol.2020.11687.

9. Fiala, O., Pitule, P., Hosek, P., Liska, V., Sorejs, O., Bruha, J., Vycital, O., Buchler, T., Poprach, A., Topolcan, O., and Finek, J. (2017). The association of miR-126-3p, miR-126-5p and miR-664-3p expression profiles with outcomes of patients with metastatic colorectal cancer treated with bevacizumab. Tumour Biol. *39*, 1010428317709283. https://doi.org/10.1177/1010428317709283.

10. Hamada, S., Satoh, K., Fujibuchi, W., Hirota, M., Kanno, A., Unno, J., Masamune, A., Kikuta, K., Kume, K., and Shimosegawa, T. (2012). MiR-126 acts as a tumor suppressor in pancreatic cancer cells via the regulation of ADAM9. Mol. Cancer Res. *10*, 3–10. https://doi.org/10.1158/1541-7786.MCR-11-0272.

11. Woźniak, O., Mierzejewski, B., and Brzoska, E. (2025). MicroRNA-126: A key regulator of angiogenesis, inflammation, and tumorigenesis - Exploring its multifaceted functions in vascular health and cancer. Biochim. Biophys. Acta Mol. Basis Dis. *1871*, 167984. https://doi.org/10.1016/j.bbadis.2025.167984.

12. Kong, Z., Wang, Y., Zhang, Y., Shan, W., Wu, J., and Wang, Q. (2020). MicroRNA-126 promotes endothelial progenitor cell proliferation and migration ability via the Notch pathway. Cardiovasc. Diagn. Ther. *10*, 490–499. https://doi.org/10.21037/cdt-20-178.

13. Han, I.B., Kim, M., Lee, S.H., Kim, J.K., Kim, S.H., Chang, J.H., and Teng, Y.D. (2016). Down-regulation of MicroRNA-126 in Glioblastoma and its Correlation with Patient Prognosis: A Pilot Study. Anticancer Res. *36*, 6691–6697. https://doi.org/10.21873/anticanres.11280.

14. Cui, H., Mu, Y., Yu, L., Xi, Y.G., Matthiesen, R., Su, X., and Sun, W. (2016). Methylation of the miR-126 gene associated with glioma progression. Fam. Cancer *15*, 317–324. https://doi.org/10.1007/s10689-015-9846-4.

15. Cappoli, N., Jenkinson, M.D., Dello Russo, C., and Dickens, D. (2022). LAT1, a novel pharmacological target for the treatment of glioblastoma. Biochem. Pharmacol. *201*, 115103. https://doi.org/10.1016/j.bcp.2022.115103.

16. Kang, S., Ughetta, M.E., Zhang, J.Y., Marallano, V.J., Sattiraju, A., Hannah, T., Wahane, S., Ramakrishnan, A., Estill, M., Tsankova, N.M., et al. (2025). Glioblastoma shift from bulk to infiltrative growth is guided by plexin-B2-mediated microglia alignment in invasive niches. Nat. Cancer *6*, 1505–1523. https://doi.org/10.1038/s43018-025-00985-4.

17. Noorani, I., de la Rosa, J., Choi, Y.H., Strong, A., Ponstingl, H., Vijayabaskar, M.S., Lee, J., Lee, E., Richard-Londt, A., Friedrich, M., et al. (2020). PiggyBac mutagenesis and exome sequencing identify genetic driver landscapes and potential therapeutic targets of EGFR-mutant gliomas. Genome Biol. *21*, 181. https://doi.org/10.1186/s13059-020-02092-2.

18. Castellani, G., Buccarelli, M., Cece, E., Offi, M., and Ricci-Vitiani, L. (2025). Aptamer-mediated delivery of therapeutic oligonucleotides in glioblastoma. Transl. Oncol. *60*, 102485. https://doi.org/10.1016/j.tranon.2025.102485.

19. Affinito, A., Quintavalle, C., Esposito, C.L., Roscigno, G., Vilardo, C., Nuzzo, S., Ricci-Vitiani, L., De Luca, G., Pallini, R., Kichkailo, A.S., et al. (2019). The Discovery of RNA Aptamers that Selectively Bind Glioblastoma Stem Cells. Mol. Ther. Nucleic Acids *18*, 99–109. https://doi.org/10.1016/j.omtn.2019.08.015.

20. Affinito, A., Quintavalle, C., Esposito, C.L., Roscigno, G., Giordano, C., Nuzzo, S., Ricci-Vitiani, L., Scognamiglio, I., Minic, Z., Pallini, R., et al. (2020). Targeting Ephrin Receptor Tyrosine Kinase A2 with a Selective Aptamer for Glioblastoma Stem Cells. Mol. Ther. Nucleic Acids *20*, 176–185. https://doi.org/10.1016/j.omtn.2020.02.005.

21. Gonda, D.D., Cheung, V.J., Muller, K.A., Goyal, A., Carter, B.S., and Chen, C.C. (2014). The Cancer Genome Atlas expression profiles of low-grade gliomas. Neurosurg. Focus *36*, E23. https://doi.org/10.3171/2012.12.focus12351.

22. Halle, B., Thomassen, M., Venkatesan, R., Kaimal, V., Marcusson, E.G., Munthe, S., Sørensen, M.D., Aaberg-Jessen, C., Jensen, S.S., Meyer, M., et al. (2016). Shift of microRNA profile upon orthotopic xenografting of glioblastoma spheroid cultures. J. Neuro Oncol. *128*, 395–404. https://doi.org/10.1007/s11060-016-2125-x.

23. Felli, N., Felicetti, F., Lustri, A.M., Errico, M.C., Bottero, L., Cannistraci, A., De Feo, A., Petrini, M., Pedini, F., Biffoni, M., et al. (2013). miR-126&126* restored expressions play a tumor suppressor role by directly regulating ADAM9 and MMP7 in melanoma. PLoS One *8*, e56824. https://doi.org/10.1371/journal.pone.0056824.

24. Marziali, G., Buccarelli, M., Giuliani, A., Ilari, R., Grande, S., Palma, A., D'Alessandris, Q.G., Martini, M., Biffoni, M., Pallini, R., and Ricci-Vitiani, L. (2017). A three-microRNA signature identifies two subtypes of glioblastoma patients with different clinical outcomes. Mol. Oncol. *11*, 1115–1129. https://doi.org/10.1002/1878-0261.12047.

25. Lin, X., Wang, H., Wu, T., Zhu, Y., and Jiang, L. (2023). Exosomes derived from stem cells from apical papilla promote angiogenesis via miR-126 under hypoxia. Oral Dis. *29*, 3408–3419. https://doi.org/10.1111/odi.14285.

26. Liu, W., Chen, H., Wong, N., Haynes, W., Baker, C.M., and Wang, X. (2017). Pseudohypoxia induced by miR-126 deactivation promotes migration and therapeutic resistance in renal cell carcinoma. Cancer Lett. *394*, 65–75. https://doi.org/10.1016/j.canlet.2017.02.025.

27. Xiang, G., and Cheng, Y. (2018). MiR-126-3p inhibits ovarian cancer proliferation and invasion via targeting PLXNB2. Reprod. Biol. *18*, 218–224. https://doi.org/10.1016/j.repbio.2018.07.005.

28. Junqueira Alves, C., Hannah, T., Sadia, S., Kolsteeg, C., Dixon, A., Wiener, R.J., Nguyen, H., Tipping, M.J., Silva Ladeira, J., Fernandes da Costa Franklin, P., et al. (2025). Invasion of glioma cells through confined space requires membrane tension regulation and mechano-electrical coupling via Plexin-B2. Nat. Commun. *16*, 272. https://doi.org/10.1038/s41467-024-55056-6.

29. Ye, L., Peng, Y., Mo, J., and Yao, Y. (2018). MiR-126 enhances VEGF expression in induced pluripotent stem cell-derived retinal neural stem cells by targeting spred-1. Int. J. Clin. Exp. Pathol. *11*, 1023–1030.

30. Dragomir, M.P., Knutsen, E., and Calin, G.A. (2022). Classical and noncanonical functions of miRNAs in cancers. Trends Genet. *38*, 379–394. https://doi.org/10.1016/j.tig.2021.10.002.

31. Chu, T.C., Twu, K.Y., Ellington, A.D., and Levy, M. (2006). Aptamer mediated siRNA delivery. Nucleic Acids Res. *34*, e73. https://doi.org/10.1093/nar/gkl388.

32. Esposito, C.L., Cerchia, L., Catuogno, S., De Vita, G., Dassie, J.P., Santamaria, G., Swiderski, P., Condorelli, G., Giangrande, P.H., and de Franciscis, V. (2014). Multifunctional aptamer-miRNA conjugates for targeted cancer therapy. Mol. Ther. *22*, 1151–1163. https://doi.org/10.1038/mt.2014.5.

33. Nuzzo, S., Roscigno, G., Affinito, A., Ingenito, F., Quintavalle, C., and Condorelli, G. (2019). Potential and Challenges of Aptamers as Specific Carriers of Therapeutic Oligonucleotides for Precision Medicine in Cancer. Cancers *11*, 1521. https://doi.org/10.3390/cancers11101521.

34. Brito, E.M., Baker, E.M., Ahye, N.M., Lieber, B.A., Hettiarachchi, S., Hollweg, M.J.M., Safar, S.B., Vanni, S., and Graham, R.M. (2025). Amino Acid Transporters in Glioblastoma: Implications for Diagnosis, Disease Monitoring, Therapeutic Targeting, and Drug Delivery. Mol. Diagn. Ther. *29*, 741–758. https://doi.org/10.1007/s40291-025-00810-9.

35. Phoenix, T.N., and Temple, S. (2010). Spred1, a negative regulator of Ras-MAPK-ERK, is enriched in CNS germinal zones, dampens NSC proliferation, and maintains ventricular zone structure. Genes Dev. *24*, 45–56. https://doi.org/10.1101/gad.1839510.

36. Lorenzo, C., and McCormick, F. (2020). SPRED proteins and their roles in signal transduction, development, and malignancy. Genes Dev. *34*, 1410–1421. https://doi.org/10.1101/gad.341222.120.

37. Al-Omari, L., Abdulsahib, W.K., Jyothi, S.R., Nayak, P.P., Chauhan, A.S., Singla, S., Sead, F.F., and Polatova, D. (2026). MicroRNA-VEGF axis in gastric cancer: a novel therapeutic frontier. Discov. Oncol. *17*, 530. https://doi.org/10.1007/s12672-026-04724-8.

38. Falchetti, M.L., D'Alessandris, Q.G., Pacioni, S., Buccarelli, M., Morgante, L., Giannetti, S., Lulli, V., Martini, M., Larocca, L.M., Vakana, E., et al. (2019). Glioblastoma endothelium drives bevacizumab-induced infiltrative growth via modulation of PLXDC1. Int. J. Cancer *144*, 1331–1344. https://doi.org/10.1002/ijc.31983.

39. Pallini, R., Ricci-Vitiani, L., Banna, G.L., Signore, M., Lombardi, D., Todaro, M., Stassi, G., Martini, M., Maira, G., Larocca, L.M., and De Maria, R. (2008). Cancer stem cell analysis and clinical outcome in patients with glioblastoma multiforme. Clin. Cancer Res. *14*, 8205–8212. https://doi.org/10.1158/1078-0432.CCR-08-0644.

40. Visconti, P., Parodi, F., Parodi, B., Casarino, L., Romano, P., Buccarelli, M., Pallini, R., D'Alessandris, Q.G., Montori, A., Pilozzi, E., and Ricci-Vitiani, L. (2021). Short tandem repeat profiling for the authentication of cancer stem-like cells. Int. J. Cancer *148*, 1489–1498. https://doi.org/10.1002/ijc.33370.

41. Castellani, G., Buccarelli, M., D'Alessandris, Q.G., De Luca, G., Ilari, R., Pedini, F., Martini, M., Mollinari, C., Tabolacci, C., Ricciardi, G., et al. (2025). DUSP8 as a regulator of glioblastoma stem-like cell contribution to tumor vascularization. J. Exp. Clin. Cancer Res. *44*, 269. https://doi.org/10.1186/s13046-025-03515-3.

42. Ricci-Vitiani, L., Pedini, F., Mollinari, C., Condorelli, G., Bonci, D., Bez, A., Colombo, A., Parati, E., Peschle, C., and De Maria, R. (2004). Absence of caspase 8 and high expression of PED protect primitive neural cells from cell death. J. Exp. Med. *200*, 1257–1266. https://doi.org/10.1084/jem.20040921.

OMTN, Volume *37*

Supplemental information

Aptamer-guided delivery of miR-126 suppresses glioblastoma stem-like cell invasion and tumor progression

Giorgia Castellani, Mariachiara Buccarelli, Alessandra Affinito, Gabriele De Luca, Ramona Ilari, Francesca Pedini, Alessandra Di Giamberardino, Mariagiovanna Ballato, Gabriele Ricciardi, Martina Offi, Cristiana Mollinari, Nadia Felli, Alessandra Boe, Giovanna Marziali, Mauro Biffoni, Maria Caffo, Gerolama Condorelli, Maurizio Martini, Quintino Giorgio D'Alessandris, and Lucia Ricci-Vitiani

Table S1. Clinical features of GBM patients and tumors.

Variable	n=58
**Age (y)**	
- median	63.0
- mean ±sd	61.8 ± 11.9
**Sex n (%)**	
- male	36 (62.1%)
- female	22 (37.9%)
**Tumor location n (%)**	
- frontal	23 (39.6%)
- temporal	18 (31%)
- parietal	11 (19%)
- occipital	3 (5.2%)
- other	3 (5.2%)
**Ki67 expression n (%)**	
- <20	19 (32.8%)
- ≥20	35 (60.3%)
- N/A	4 (6.9%)
**MGMT status n (%)**	
- Methylated	30 (51.7%)
- Unmethylated	11 (19%)
- N/A	17 (29.3%)
**KPS n (%)**	
- **≤70**	18 (31%)
- **>70**	40 (69%)

Abbreviations: MGMT, O6-methylguanine-DNA-methyltransferase; KPS, Karnofsky performance status.

Table S2. Scheme of the intracranial implantation of GSCs into immunocompromised mice and aptamer-based treatment protocol.

**Group**	**n**	**Treatment (Dose)**	**Schedule**	**Administration method**
**I**	10	saline	Three times/week (Three Weeks)	i.v. (tail vein)
**II**	10	A40s (2500 pmol)	Three times/week (Three Weeks)	i.v. (tail vein)
**III**	10	A40s-miR-126-3p (2500 pmol)	Three times/week (Three Weeks)	i.v. (tail vein)

A

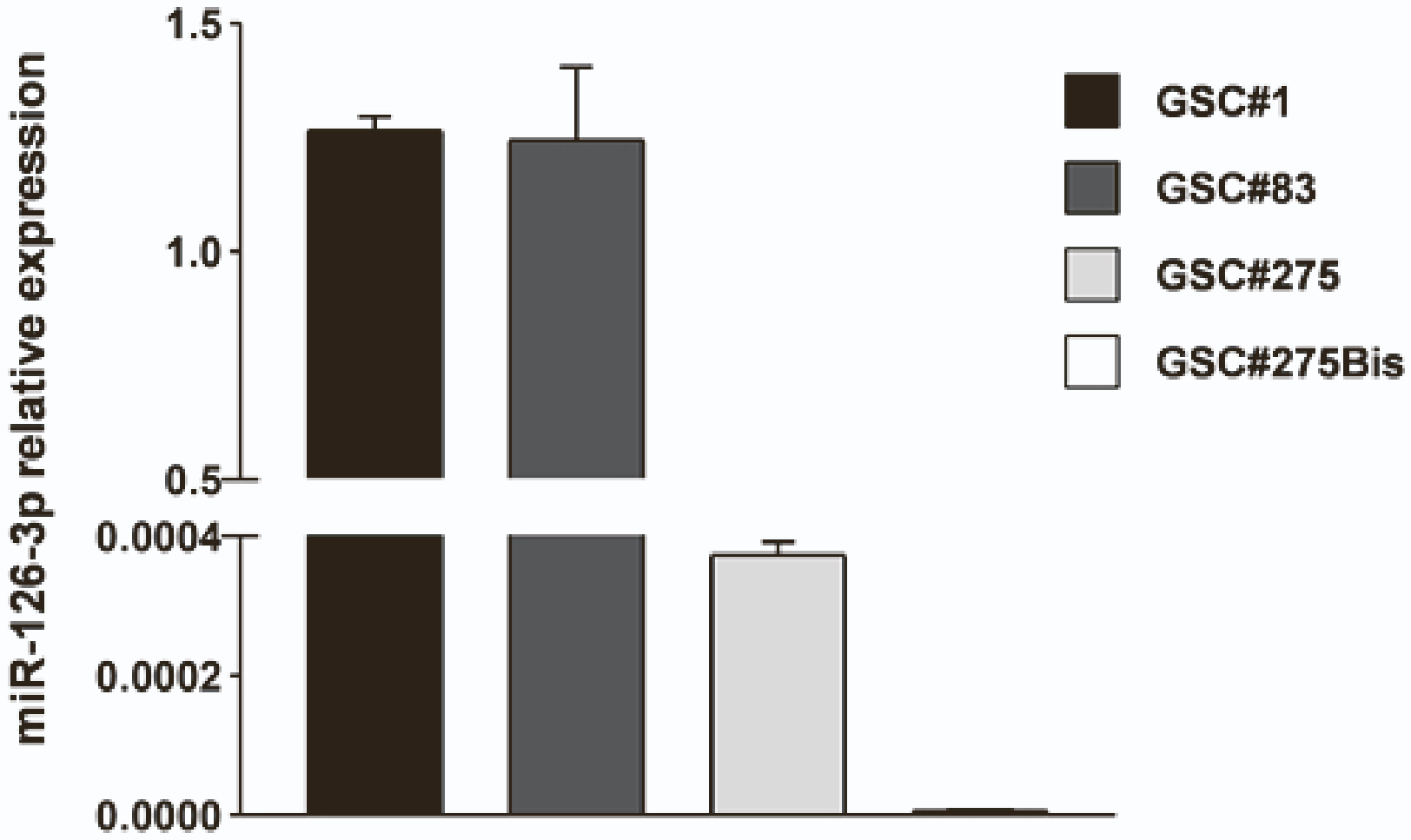

B

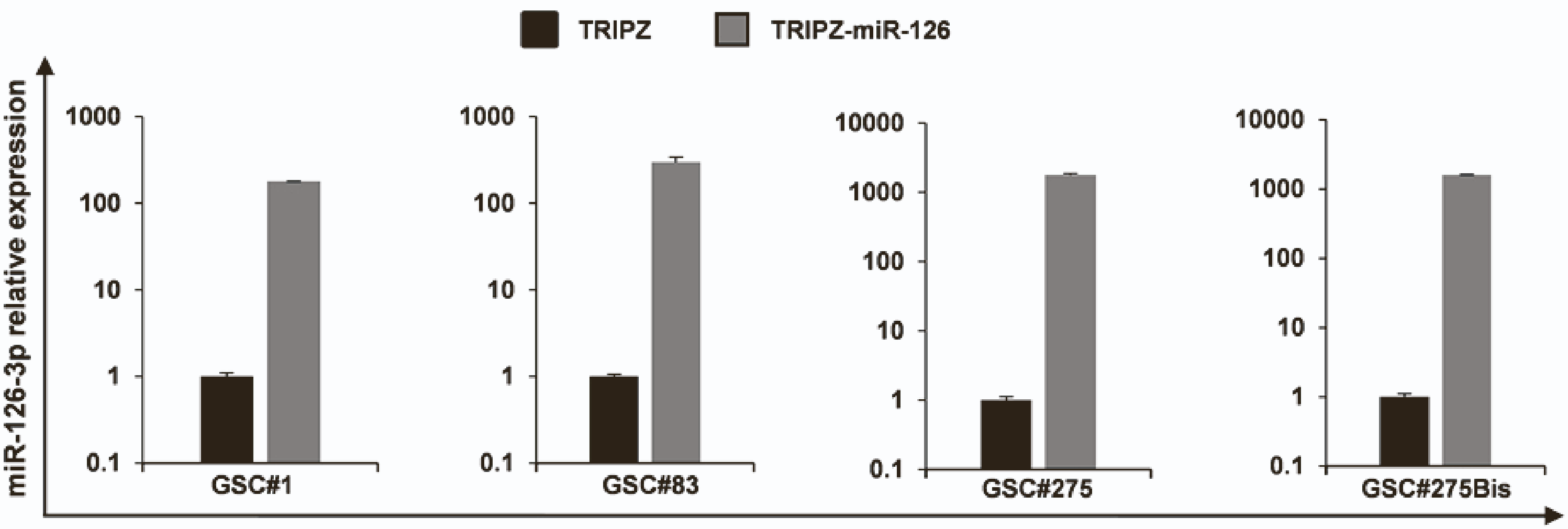

C

	GSC#1		GSC#83		GSC#275		GSC#275Bis	
	TRIPZ	TRIPZ-miR126	TRIPZ	TRIPZ-miR126	TRIPZ	TRIPZ-miR126	TRIPZ	TRIPZ-miR126
LAT1	27.6 ± 0.1	24.4 ± 0.02	25.6 ± 0.04	27.2 ± 0.1	25.8 ± 0.01	26.8 ± 0.3	25.0 ± 0.07	26.1 ± 0.2
PLXNB2	29.5 ± 0.1	26.9 ± 0.1	34.0 ± 0.2	33.9 ± 0.2	29.3 ± 0.1	30.0 ± 0.03	35.0 ± 0.08	35.7 ± 0.06
SPRED1	26.7 ± 0.09	25.7 ± 0.2	26.8 ± 0.06	26.6 ± 0.08	28.4 ± 0.3	28.1 ± 0.2	26.9 ± 0.01	27.7 ± 0.09
GAPDH	20.9 ± 0.02	18.9 ± 0.02	22.3 ± 0.2	21.6 ± 0.07	19.9 ± 0.6	19.2 ± 0.6	20.8 ± 0.2	20.9 ± 0.004

Figure S1. miR-126-3p basal expression on selected GSC lines and effects of miR-126 restoration on its targets. (A) RT-PCR analysis of miR-126-3p in the 4 selected GSC lines (GSC#1, GSC#83, GSC#275 and GSC#275Bis). **(B)** RT-PCR analysis of miR-126-3p in the 4 selected GSC lines transduced with either TRIPZ or TRIPZ-miR-126 vectors. Samples were run in duplicate. Data were normalized to the RNU6B expression in the corresponding samples. **(C)** Raw Ct values from RT-PCR analysis of LAT1, PLXNB2, and SPRED1 in GSC#1, GSC#83, GSC#275, and GSC#275Bis cells transduced with TRIPZ or TRIPZ-miR-126 vectors. Data are presented as mean ± SD.

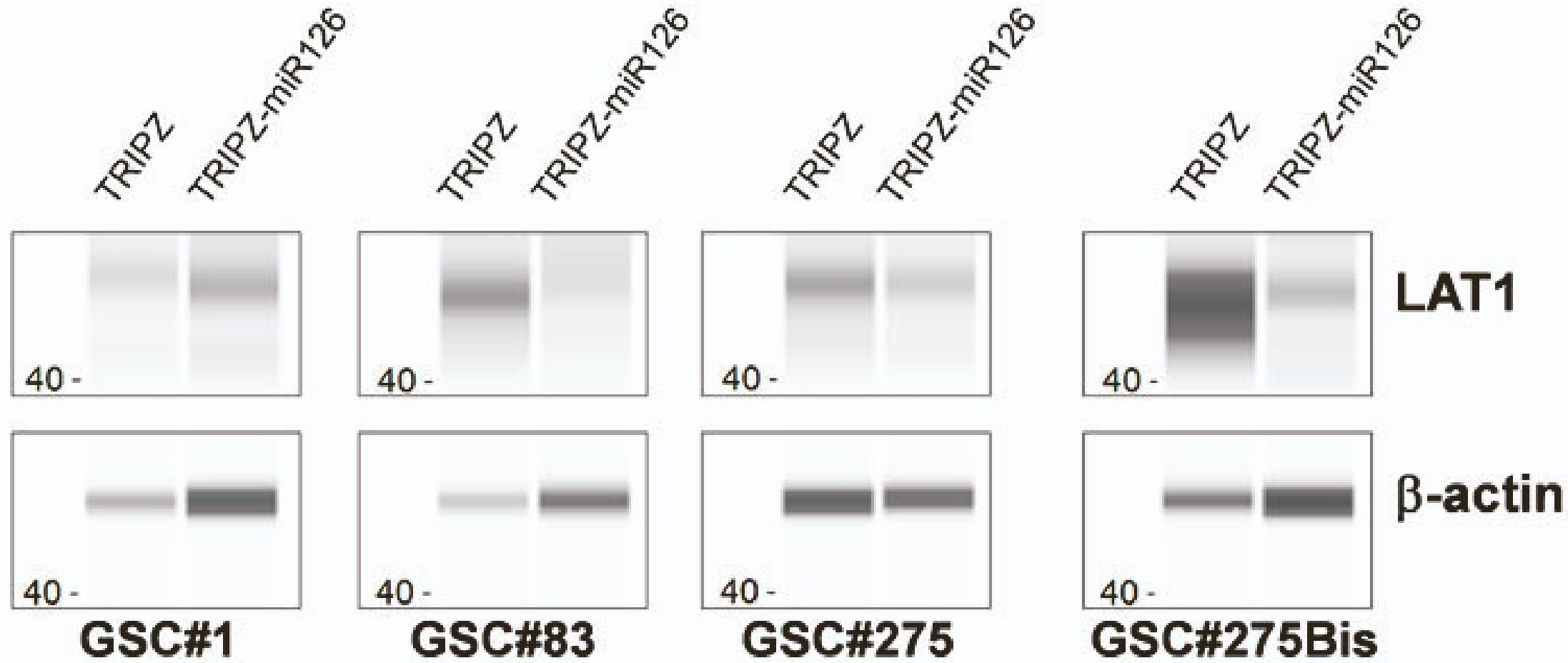

	GSC#1 TRIPZ	GSC#1 TRIPZ-miR126	GSC#83 TRIPZ	GSC#83 TRIPZ-miR126	GSC#275 TRIPZ	GSC#275 TRIPZ-miR126	GSC#275Bis TRIPZ	GSC#275Bis TRIPZ-miR126
**LAT1**	1359,1	3423,7	9855,6	1652,8	6467,4	3049,2	31136,2	8314,0
**β-actin**	3539,7	8564,3	2521,4	5071,3	8691,9	7165,4	5614,1	10309,7

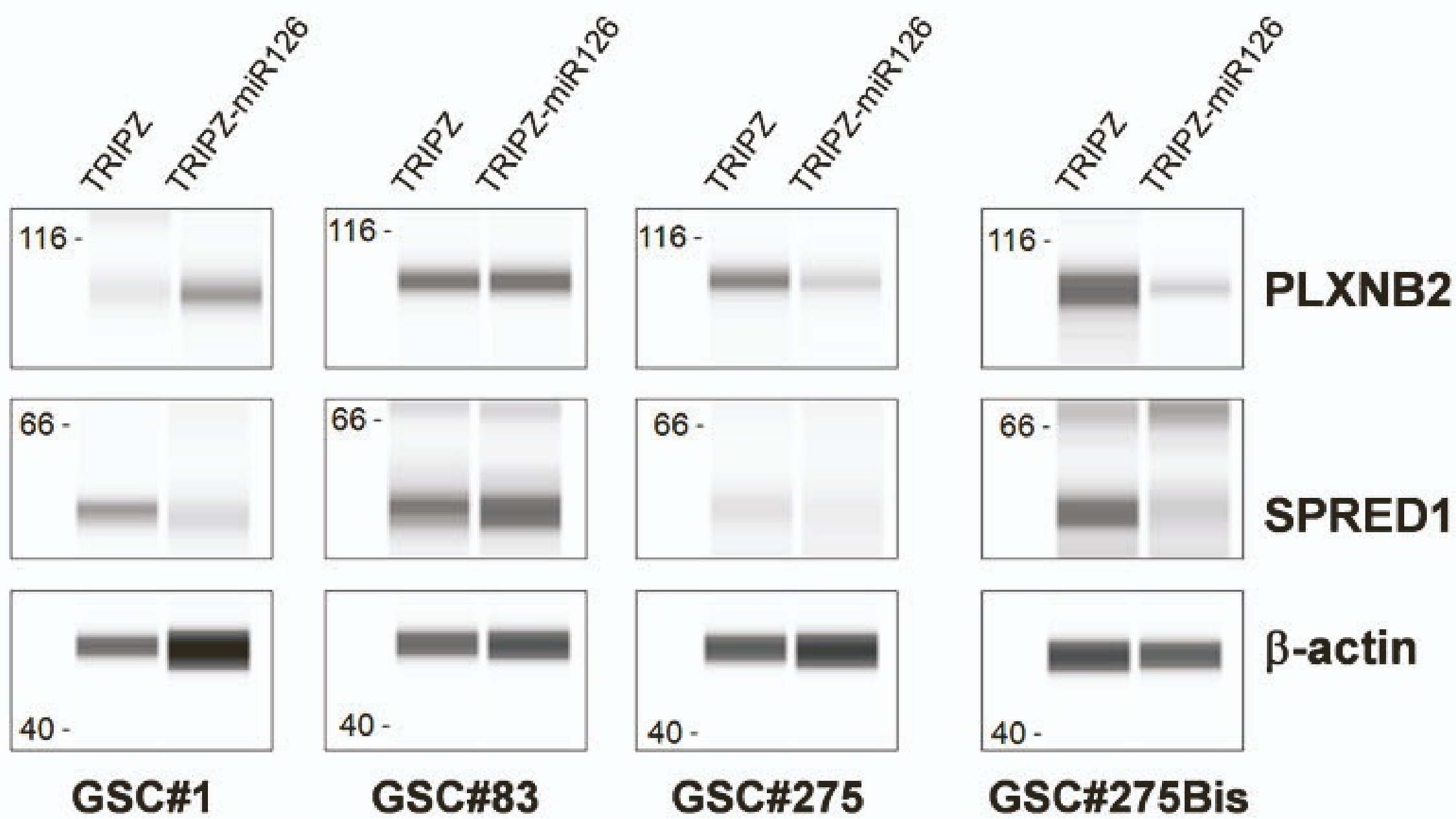

	GSC#1 TRIPZ	GSC#1 TRIPZ-miR126	GSC#83 TRIPZ	GSC#83 TRIPZ-miR126	GSC#275 TRIPZ	GSC#275 TRIPZ-miR126	GSC#275Bis TRIPZ	GSC#275Bis TRIPZ-miR126
**PLXNB2**	25317,7	40311,9	78489,9	77913,3	42284,9	25217,6	99457,3	33929,8
**SPRED1**	9767,3	9822,6	22055,3	23658,0	9618,5	8428,4	12240,2	5456,1
**β-actin**	69303,1	212044,7	157769,3	201318,1	178311,5	261886,2	248778,7	196959,4

Figure S2. miR-126 restoration induces a significant decrease in the level of target proteins. WES full-length gel image of LAT1, PLXNB2 and SPRED1 expression in the 4 selected GSC lines (GSC#1, GSC#83, GSC#275 and GSC#275Bis) transduced with either TRIPZ or TRIPZ-miR-126 vectors. Protein expression was quantified using AUC (area under the curve) measurements generated using Compass Software. Signal intensity was normalized to the β-actin expression in the corresponding sample. Representative images from two independent experiments are shown.

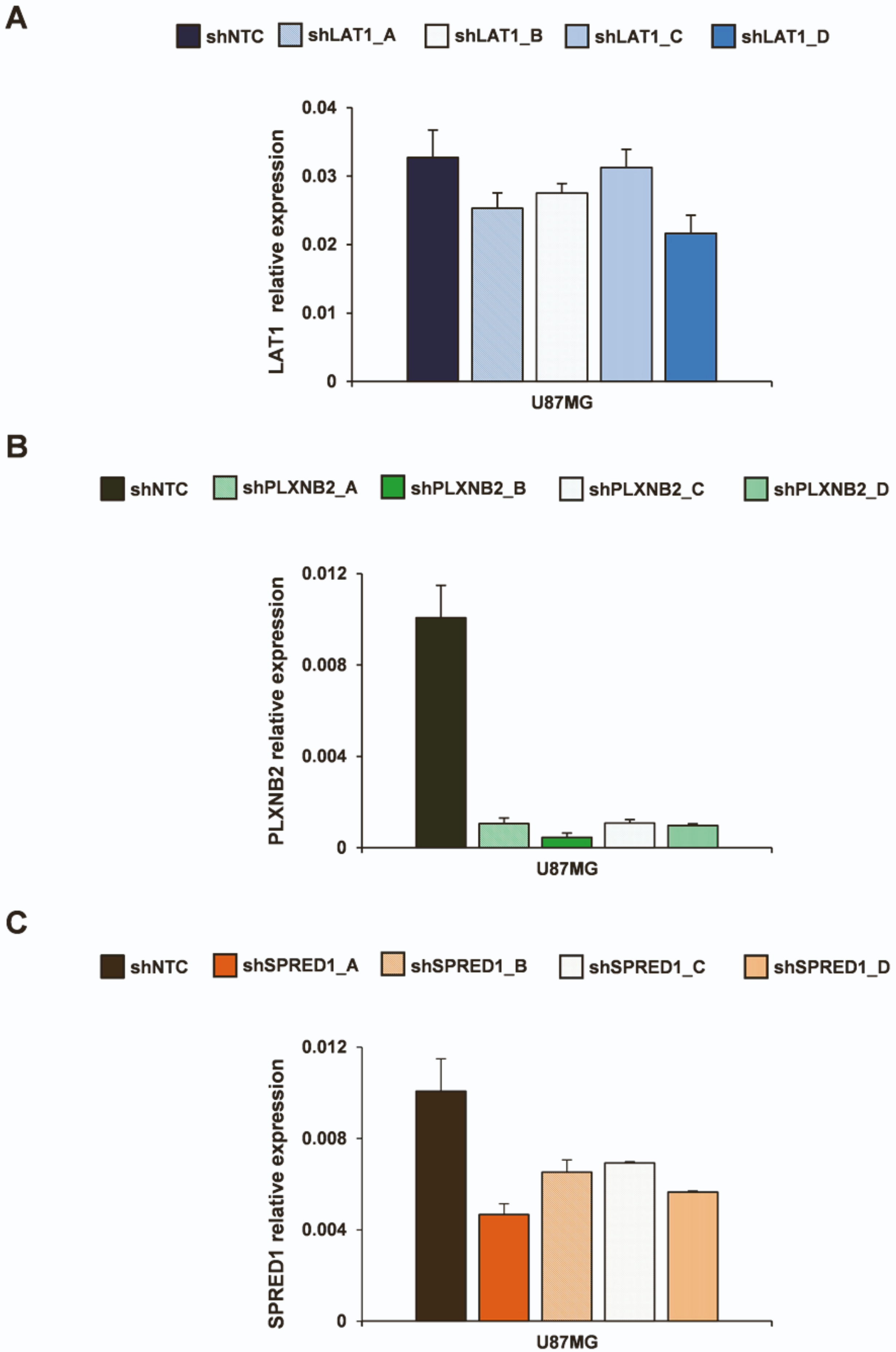

Figure S3. Selection of the most effective sh-RNA vectors on target silencing in U87MG glioma cell line. RT-PCR analysis of LAT1 **(A)**, PLXNB2 **(B)**, and SPRED1 **(C)** in U87MG cells transduced with shNTC or four shRNA specific vectors (A–D). Samples were run in duplicate, and data were normalized to GAPDH expression.

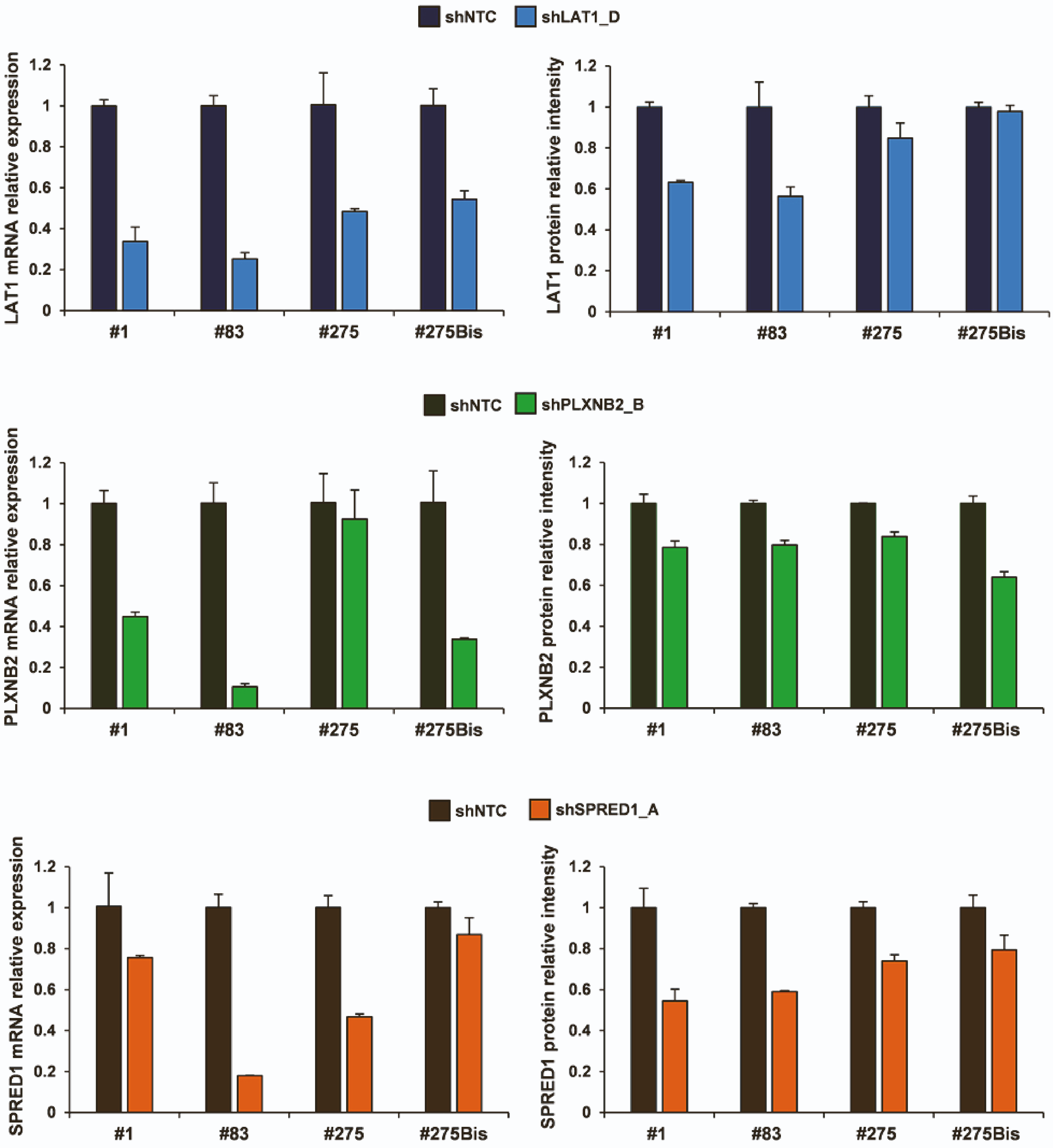

Figure S4. Effect of the selected shRNA vectors on target silencing. RT-PCR analysis of LAT1, PLXNB2, and SPRED1 in GSC#1, GSC#83, GSC#275, and GSC#275Bis cells transduced with shNTC or the most effective shRNA vector. Samples were run in duplicate, and data were normalized to GAPDH expression (*left panels*). WES analysis of LAT1, PLXNB2, and SPRED1 in GSC#1, GSC#83, GSC#275, and GSC#275Bis cells transduced with shNTC or the most effective shRNA vector. Protein levels were quantified using AUC measurements and normalized to β-actin expression (*right panels*).

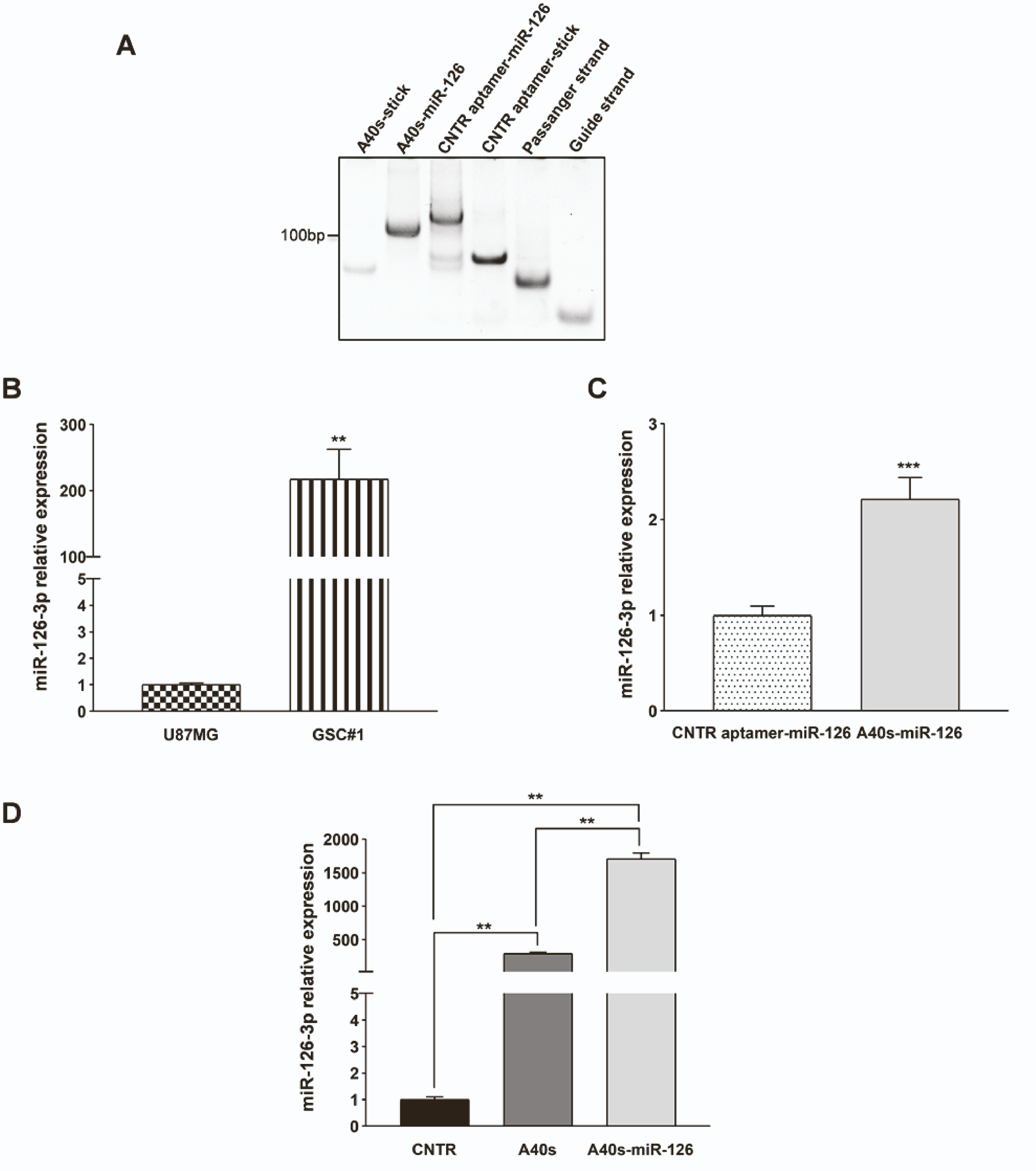

Figure S5. Successfully annealing of the A40s-miR-126-3p chimera. (A) Non-denaturing gel electrophoresis analysis reporting correct annealing of the A40s-miR126-3p chimera conjugate. **(B)** Relative miR-126-3p levels assessed by RT-PCR after incubation of GSC#1 with A40s-miR126 chimera and compared to U87MG as an EphA2 negative cell line. Bars report mean of three independent experiments ± SD. **p ≤ 0.01(Student's t test). **(C)** Relative miR-126-3p levels assessed by RT-PCR after incubation of GSC#1 with A40s-miR126 chimera and compared with an unrelated sequence-based chimera as a control. Bars report mean of three independent experiments ± SD. ***p ≤ 0.001 (Student's t test). **(D)** RT-PCR for miR-126-3p expression of no treated GSC#1 (CNTR), GSC#1 incubated with A40s-aptamer alone (A40s) or A40s-miR126-3p chimera (A40s-miR126).

A

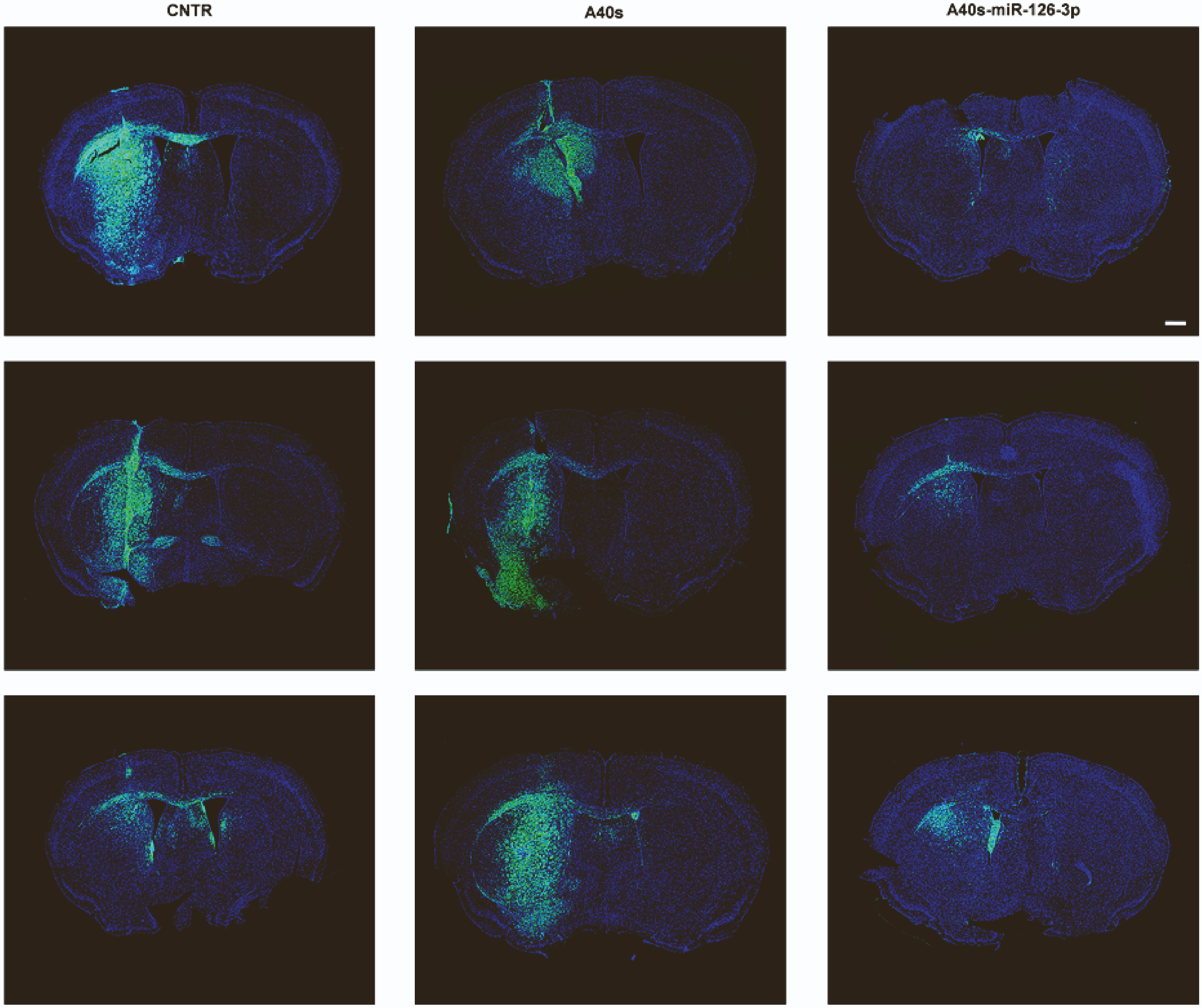

B

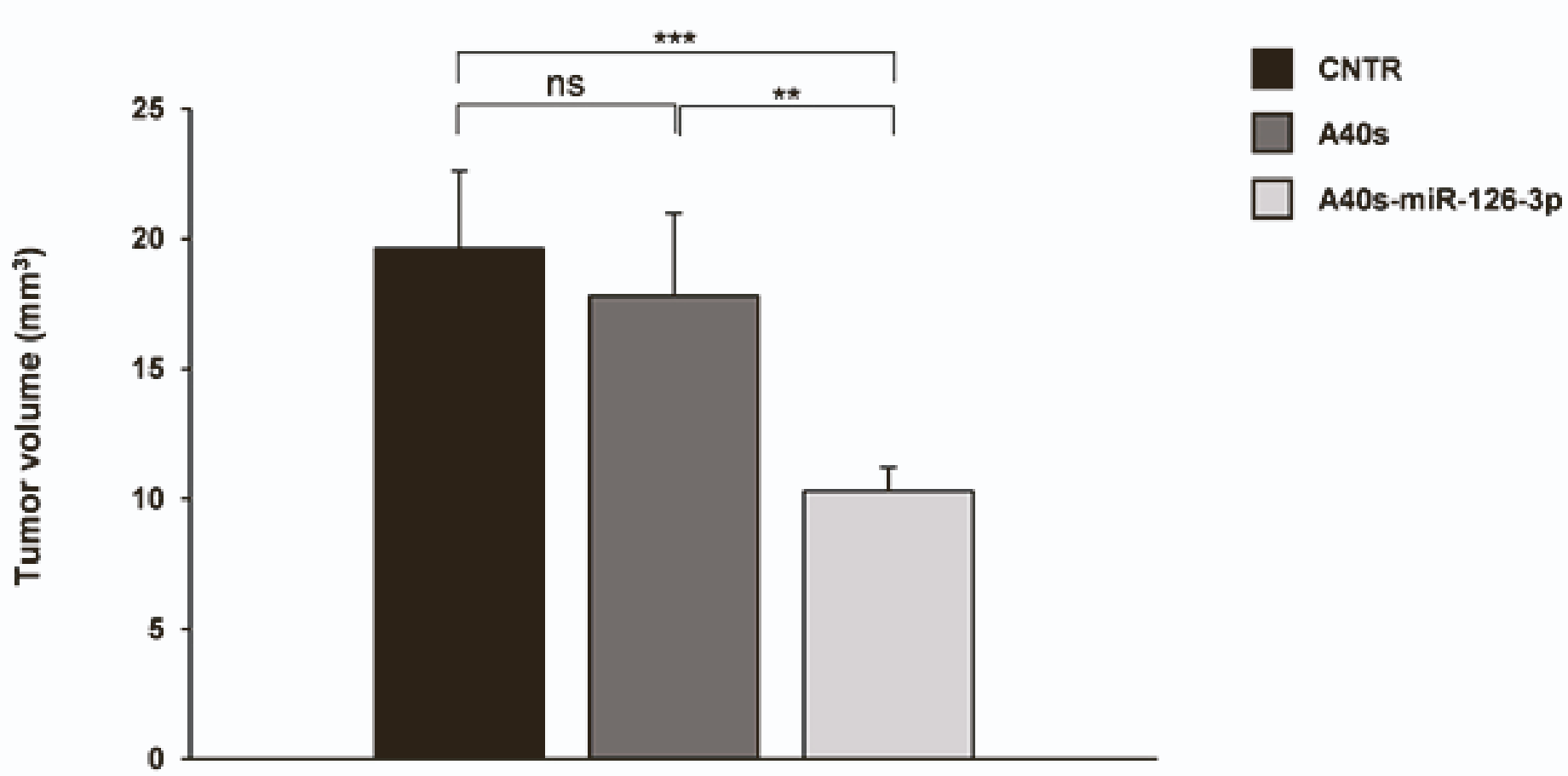

Figure S6 The in vivo effects of aptamer A40s-miR-126-3p on tumor xenografts in mice. **(A)** Images of coronal mouse brain sections at 8 weeks after the intracranial injection of GFP⁺ GSC#1. One week after grafting mice were randomly assigned to three groups: saline treated (CNTR), treated with A40s-aptamer alone (A40s) or A40s-miR-126-3p chimera (A40s-miR-126-3p). Free floating brain slices were counterstained with DAPI (blue). Scale bar: 500 µm. **B** Graph showing the tumor volume for the intracranial tumors after 8 weeks from the injection of GSCs. ns (not significant), **p<0.01, ***p<0.001 based on Student's t test
